# Enhancing photo-reduction quantum efficiency using quasi-type II core/shell quantum dots†

**DOI:** 10.1039/c6sc00192k

**Published:** 2016-03-02

**Authors:** Yanyan Jia, Jinquan Chen, Kaifeng Wu, Alex Kaledin, Djamaladdin G. Musaev, Zhaoxiong Xie, Tianquan Lian

**Affiliations:** a Department of Chemistry, Emory University Atlanta Georgia 30322 USA tlian@emory.edu; b State Key Laboratory of Physical Chemistry of Solid Surfaces, Department of Chemistry, College of Chemistry and Chemical Engineering, Xiamen University Xiamen 361005 China

## Abstract

Quantum confined semiconductor nanocrystals have emerged as a new class of materials for light harvesting and charge separation applications due to the ability to control their properties through rational design of their size, shape and composition. We report here a study of enhancing the quantum yield of methyl viologen (MV^2+^) photoreduction using colloidal quasi-type II CdSe/CdS core/shell quantum dots (QDs). The steady-state quantum yield of MV^+^˙ radical generation, in the presence of thiols as sacrificial donors, increased monotonically with the CdS shell thickness within the studied thickness regime (0–4.7 CdS monolayers). Using ultrafast transient absorption and time-resolved photoluminescence decay spectroscopy, we found that both the rates of electron transfer from the QD to MV^2+^ and the subsequent charge recombination in QD^+^–MV^+^˙ complexes decreased exponentially with the shell thickness, consistent with calculated 1S electron and hole densities at the QD surfaces, respectively. Interestingly, the hole transfer rate remained relatively independent of shell thickness, likely due to a cancellation of the reduction of hole transfer coupling strength with the increased number of hole acceptor ligands on the QD surface at larger shell thickness. As a result, with increasing CdS shell thickness, the charge recombination loss decreases, enhancing the photoreduction quantum efficiency. This novel approach for improving photoreduction quantum efficiency should be applicable to many type II and quasi-type II core/shell quantum dots.

## Abstract 2

Enhancing photoreduction quantum yield in core/shell QDs through an unexpectedly fast increase of hole removal rate with shell thickness.

## Graphical abstract

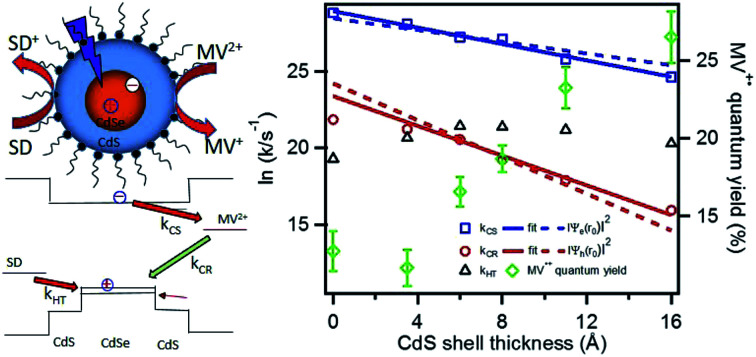

## Introduction

**Scheme 1:**
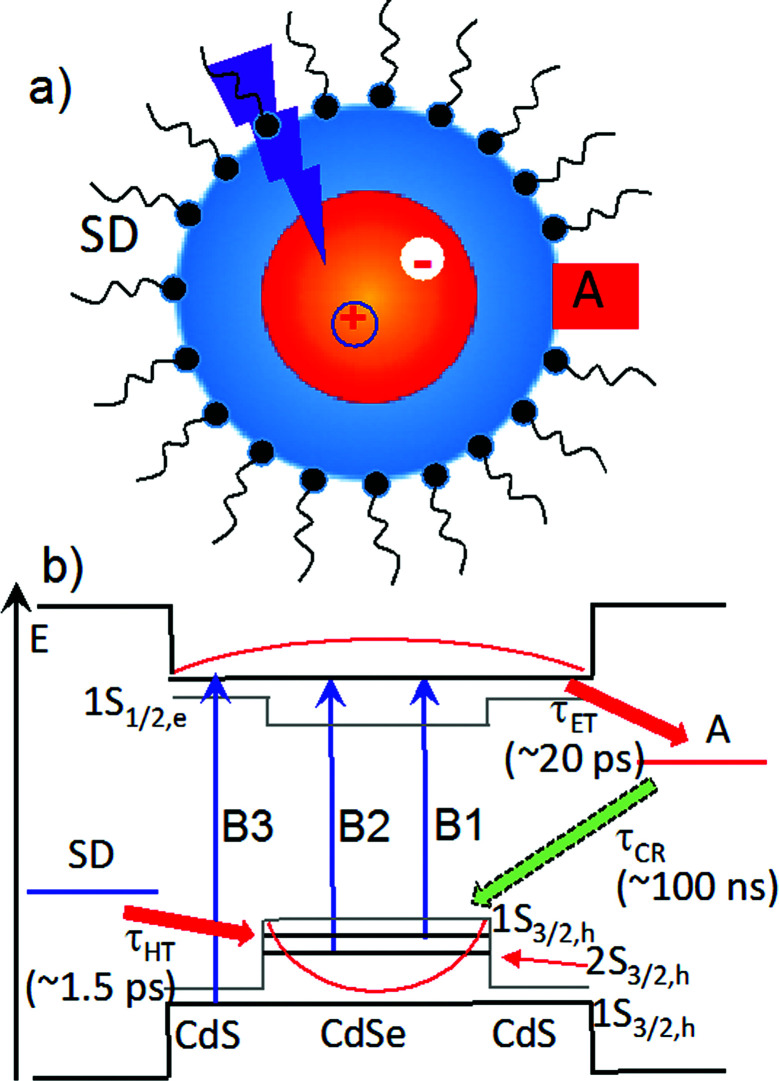
Sacrificial donor–QD–acceptor (D–QD–A) complex. (a) Schematic structure of D–QD–A complex with D as the surface capping ligand. (b) Relevant energy level diagrams (thick solid line), bulk band alignment (thin grey line), charge transfer processes (red and green arrows) and transitions (blue vertical arrows) in a quasi-type II CdSe/CdS core/shell QD. B1, B2 B3 are transitions to the lowest energy CB electron level (1S_1/2,e_) from the 1S_3/2,h_ (CdSe), 2S_3/2,h_ (CdSe), and 1S_3/2,h_ (CdS), respectively. Forward electron transfer to acceptor (*τ*_ET_) and hole filling by sacrificial electron donor (*τ*_HT_) compete with charge recombination (*τ*_CR_). Time constants for these processes for 4.7 ML CdSe/CdS QDs (with A = MV^2+^ and SD = MUA) are shown inside the parenthesis.

In recent years, quantum confined semiconductor nanomaterials, such as quantum dots (QDs), nanorods and nanoplatelets, have received intense interest as light harvesting and charge separating materials for solar energy conversion.^1–6^ These novel materials offer many unique properties, including size-dependent absorption, large extinction coefficients over a broad spectral range, long exciton lifetimes, multiple exciton generation, enhanced photo-stability, and facile control of the spatial distribution of electrons and holes through size, composition and shape (*i.e.* wave function engineering).^7–11^ As shown in Scheme 1, in a typical photoreduction process, the conduction band (CB) electron in an excited light absorber is transferred to the electron acceptor (with a time constant of *τ*_ET_) and the valence band (VB) hole to the (sacrificial) electron donor (*τ*_HT_). These desirable forward processes compete with electron–hole recombination both within the nanomaterials in the excitonic state and across the interface in charge separated states (*τ*_CR_). It was shown previously that the rate of electron and hole transfer from QDs depends on electron and hole densities at the QD surface,^12^ which suggests the possibility of control these rates through wave function engineering. This concept was demonstrated in a study of core/shell QDs with type II band alignment, in which the shell-localized 1S electrons and core localized 1S holes were shown to facilitate ultrafast electron transfer to adsorbed acceptors while simultaneously retarding the charge recombination process.^13^ The combination of efficient charge separation and slow recombination in quasi-type II and type II QDs suggest that they could be ideal materials for delivering electrons to catalysts or redox mediators.^14–16^

In this paper, we report a novel approach for enhancing the quantum efficiencies of photoreduction using colloidal quasi-type II CdSe/CdS QDs (Scheme 1). Methylviologen (MV^2+^) was chosen for this study because it is a well-known one-electron redox mediator for many photocatalytic reactions,^17–21^ and ultrafast electron transfer from semiconductor nanomaterials to MV^2+^ has been observed.^21–24^ We demonstrated that the quantum yield of MV^2+^ photo-reduction increased with CdS shell thickness from 0 (core only) to 4.7 monolayers of CdS. Using ultrafast transient absorption and time-resolved photoluminescence decay spectroscopy, we found that with increasing shell thickness, both charge separation and recombination rates decreased exponentially, which agreed well with computed change of surface electron and hole densities. Surprisingly, the hole removal rate to sacrificial electron donors remained nearly constant, which can be attributed to an increase of the surface area for binding of sacrificial electron donors. Therefore, with increasing shell thickness, the photoreduction quantum efficiency increases due to the reduction in the recombination loss. Our finding may provide a new way for optimizing photoreduction quantum efficiency using semiconductor nanostructures.

## Results and discussion

### Characterization of QDs

**Fig. 1:**
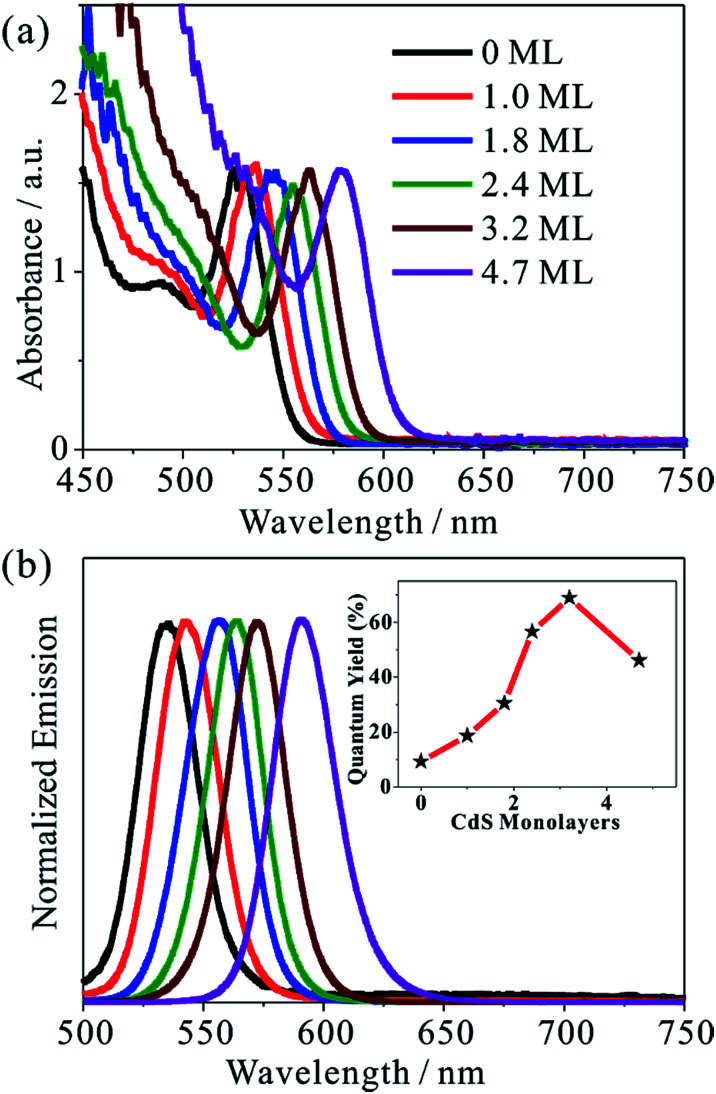
Absorption and PL spectra of CdSe/CdS QDs. (a) Normalized UV-vis absorption and (b) PL spectra of CdSe/CdS QDs with indicated MLs of CdS in chloroform solution. Inset in (b): the PL quantum yield as a function of the CdS shell thickness.

We synthesized five different CdSe/CdS QDs using 2.8 nm diameter CdSe seed and the TEM images of these samples are shown in Fig. S1.† From the diameter differences of the CdSe seed and CdSe/CdS core/shell QDs, the average shell thicknesses were determined to be 1.0, 1.8, 2.4, 3.2 and 4.7 monolayer (ML) of CdS (0.34 nm per CdS layer) for samples CdSe/CdS #1 to CdSe/CdS #5 (see Table S1 in ESI†).^25^ For convenience, we label these QDs according to the number of CdS shell layers (with 0 ML for CdSe core only QDs). Fig. 1a and b show the UV-vis absorption and photoluminescence (PL) spectra, respectively, of above QDs and the CdSe seed. With increasing shell thicknesses, the first excitonic absorption (emission) peaks red-shift from 525 nm (535 nm) for CdSe seed to 585 nm (603 nm) for the 4.7 ML CdSe/CdS QD. The red-shifting of both absorption and PL peaks can be attributed to electron wave function delocalization from CdSe core into CdS shell, which lowers the electron confinement energy.^12,26^ As shown in the Fig. 1b inset, the CdS shell also suppresses the deep trap emission of the CdSe core and increases the quantum yield from ∼10% in the CdSe core to ∼70% in CdSe/CdS (3.2 MLs) by passivating surface defects.^27^ A further increase of CdS shell thickness leads to a reduction of the PL quantum yield, which has been attributed to an increase in the lattice-mismatch-induced defects.^12^

### Steady-state MV^2+^ photo-reduction quantum efficiencies

MUA-capped water soluble CdSe/CdS were prepared following a similar protocol reported before^28^ (see ESI† in detail) and used in the MV^2+^ photo-reduction experiments. The experiments were performed by mixing above CdSe/CdS QDs, MV^2+^ (5.0 mM) and sacrificial electron donors in anaerobic aqueous solutions. 50 mM 3-mercaptopropionic acid (MPA) was used as excess sacrificial electron donors. The QD concentrations were adjusted to ensure that all solutions had the same absorbance at the illumination wavelength (0.3 OD at 405 nm).

**Fig. 2:**
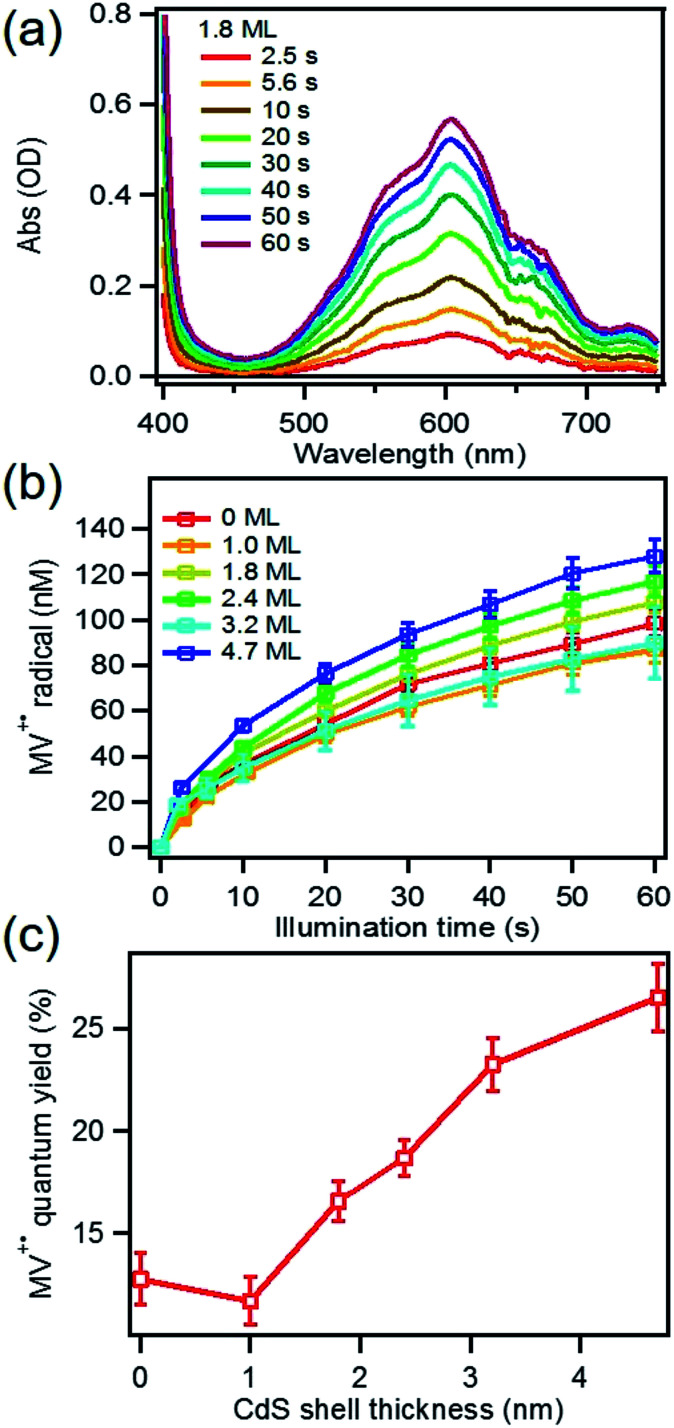
Static state photoreduction of MV^2+^ using CdSe/CdS core/shell QDs (a) UV-vis difference spectra (after–before illumination) of a solution containing 1.8 ML CdSe/CdS QDs, 5 mM MV^2+^ and 50 mM MPA under 405 nm (23.8 mW) illumination; (b) MV^+^˙ radical generation kinetics using QDs with different shell thickness under conditions described in (a); (c) initial quantum yields of MV^+^˙ radical generation as a function of shell thickness.

Under these conditions, MV^+^˙ radicals formed quickly under 405 nm illumination, as indicated by the growth of a distinct 605 nm band in the difference spectra shown in Fig. 2a. Complete sets of steady state UV-vis difference spectra for all CdSe/CdS QDs are shown in Fig. S2 of ESI.† The amount of MV^+^˙ radicals was calculated to obtain the radical formation kinetics using the extinction coefficient in the literature (13 700 ± 300 M^−1^ cm^−1^ at 605 nm).^29^ The photon-reduction quantum yields of MV^2+^ is defined in eqn (1).1
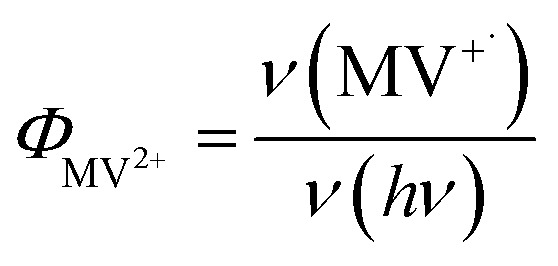
where *ν*(MV^+^˙) is the MV^+^˙ radical cation generation rate (which is the slope of MV^+^˙) *vs.* time plots shown in Fig. 2b and *ν*(*hν*) is the photon absorption rate of the reaction solution, respectively. Due to the consumption of the electron and/or hole acceptors the rate decreased slowly with time. For this reason, only the initial quantum yields were calculated and shown in Fig. 2c. Under our experimental conditions, CdSe seed showed a 12.7 ± 1.2% initial quantum yield of MV^2+^ photo-reduction which is in good agreement with previous reported values at similar conditions.^21^ With increased CdS shell thickness, the quantum yield of MV^2+^ photo-reduction showed a systematically increasing from 12.7 ± 1.2% for CdSe seed to 26.5 ± 1.7% for 4.7 ML CdSe/CdS QD as listed in Table 1. In these systems, the overall MV^+^˙ radical generation quantum yield should depend on the rates of charge separation, charge recombination, and hole-filling processes. Next, we used time-resolved transient absorption and PL decay spectroscopy to elucidate the mechanism of the increased MV^2+^ photo-reduction performances as CdS shell thickness increasing.

**Table 1:** Measured and estimated yields and time constants: charge separation (*τ*_CS_), charge recombination (*τ*_CR_) and hole transfer (*τ*_HT_)

Sample	*Φ*_MV_ (%)	*τ*_CS_ (ps)	*τ*_CR_ (ns)	*τ*_HT_ (ns)
0 ML	12.7 ± 1.2	0.3 ± 0.1	0.5 ± 0.1	4.2 ± 0.2
1.0 ML	11.7 ± 1.1	0.6 ± 0.2	0.6 ± 0.1	1.1 ± 0.2
1.8 ML	16.6 ± 1.0	1.5 ± 0.1	1.8 ± 0.2	0.5 ± 0.1
2.4 ML	18.7 ± 0.9	1.7 ± 0.1	4.4 ± 0.3	0.5 ± 0.1
3.2 ML	23.2 ± 1.3	6.3 ± 0.3	18 ± 1	0.6 ± 0.2
4.7 ML	26.5 ± 1.7	20 ± 1	100 ± 2	1.5 ± 0.3

### Electron and hole dynamics in free CdSe/CdS QDs

**Fig. 3:**
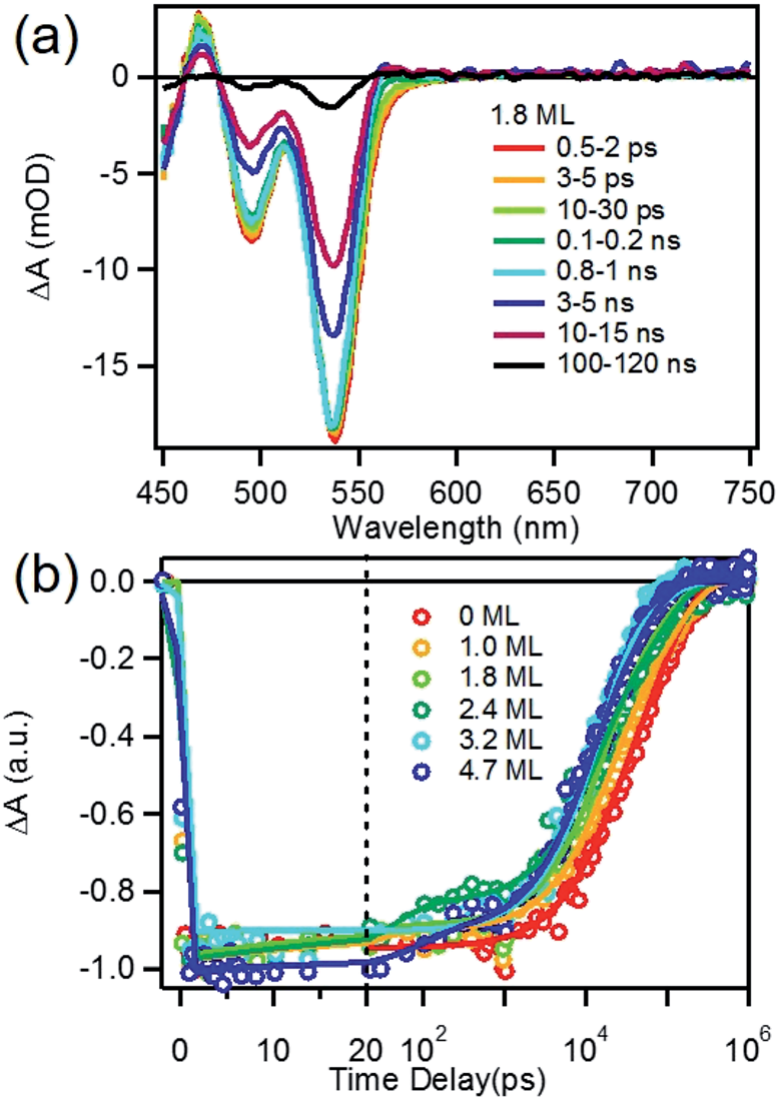
Transient absorption spectra and kinetics of TOP-capped CdSe/CdS QDs in chloroform. (a) Average transient absorption spectra of free CdSe/CdS (1.8 ML) QDs at indicated delay time windows after 530 nm excitation. (b) Comparison of 1S (B1) exciton bleach recovery kinetics in QDs with 0, 1.0, 1.8, 2.4, 3.2 and 4.7 MLs of CdS shells.

We first investigated free trioctylphosphine (TOP)-capped QDs in chloroform by transient absorption (TA) spectroscopy with 530 nm excitation. The transient absorption spectra of free QDs with different shell thicknesses are shown in Fig. 3 (1.8 ML) and S3† (0, 1.0, 1.8, 2.4, 3.2 and 4.7 MLs). These spectra were obtained at low excitation photon fluence (5.0 × 10^−5^ J cm^−2^) to ensure negligible contributions of multi-exciton states.^30^ For CdSe core only QDs, the TA spectra showed a long-lived bleach of the 1S excitonic absorption peak (1S_3/2h_–1S_1/2e_ transition or B1 in Scheme 1b) at 538 nm and a higher energy exciton band (2S_3/2h_–1S_1/2e_ transition or B2 in Scheme 1b) at 495 nm.^31^ With increasing CdSe shell thickness, these peaks shift to lower energy and the energy difference between them becomes smaller, indicating a decrease in the confinement energy for both electrons and holes in CdSe core.^31^ In addition, in 4.7 ML CdSe/CdS QDs, a strong bleach at 475 nm was also observed and can be attributed to the lowest energy transitions in the CdS shell (1S_3/2h_–1S_1/2e_ transition in CdS or B3 in Scheme 1b). The B3 bleach gradually increases in amplitude and redshifts with increasing shell thickness (Fig. S3†), consistent with this assignment. As shown in Fig. S4,† a comparison B1, B2 and B3 bleach kinetics for each core/shell QDs show that they are identical. Since these bleaches are assigned to the state filling of the conduction band (CB) electron level,^16,32^ the instantaneous formation of both CdSe and CdS bleaches after 530 nm excitation (promoting an electron from the CdSe VB) indicates that the electron wave function extends from the CdSe core into the CdS shell, consistent with a quasi-type II electronic structure.^32,33^

A comparison of 1S exciton bleach recovery kinetics for QDs of different shell thickness is shown in Fig. 3b. In all samples, the bleach recovers by <15% within 1 ns in all core/shell QDs, confirming that the dominance of single exciton states.^12^ These kinetics can be fitted by an instrument response limited rise and three exponential decay with a half-life from 28 ns in CdSe core only QDs to 9.6 ns in 4.7 ML CdSe/CdS (Table S2A†). PL decays of these QDs are shown in Fig. S10† and the parameters of multi-exponential fits to these decay kinetics are listed in Table S7.† PL decay of CdSe core shows a multi-exponential decay with a half-life of 15.6 ns. However, CdSe/CdS QDs show a slightly smaller PL half-life times (Table S7†) for 1.0 to 4.7 ML CdS shell. Both the trends of 1S exciton bleach recovery (a measure of 1S electron lifetime) and the PL decay (reflecting both the 1S electron and hole lifetimes) are inconsistent with the expected trend of radiative electron–hole recombination time in quasi-type II core/shell QDs which is reported to increase with shell thickness due to electron–hole spatial overlap.^34^ This result suggests that the electron and hole decay dynamics contain significant contribution of non-radiative decay process, which is consistent with the non-unity quantum yield in these QDs (Fig. 1b inset). In order to determine the contributions of nonradiative decay pathways to the electron and hole dynamics, we compared 1S bleach TA signal with PL decay for each CdSe/CdS QD (Fig. S11†). The comparison shows that in all samples, the PL decay is much faster than the 1S electron bleach recovery kinetics. PL decay kinetics in 1.0, 1.8 and 2.4 ML CdSe/CdS QDs do not match with 1S bleach recovery kinetics until ∼100 ns and these kinetics start to agree with each other after about 10 ns for 3.2 and 4.7 ML CdSe/CdS QDs. There is a fast decay component with a decay time constant of ∼2–3 ns in the PL decay kinetic (Table S7†) that is absent in the transient 1S bleach kinetics (Table S2A†) for all the QDs. Since 1S exciton bleach follows the electron kinetics, the fast PL decay component can be attributed to hole trapping in these QDs. When CdS shell thickness reaches 3.2 ML and above, the PL decay agree better with 1S bleach recovery (compared to thinner shells). This result suggests that with increasing shell thickness, the surface hole trap states of CdSe core are better passivated and the contribution to the hole trapping deceases, and the contribution of electron–hole radiative recombination and PL quantum yield increases.

We next examined the effect of MUA capping ligand on the carrier dynamics in MUA-capped water soluble CdSe/CdS QDs used in the steady-state MV^2+^ photoreduction described above. The transient spectra and 1S exciton bleach recovery kinetics of these samples are shown in Fig. S5 and S6 in ESI.† Similar to QDs in chloroform, strong B1 and B2 band bleach of the CdSe core and the B3 bleach of CdS shell were observed in all the water soluble QDs after 530 nm excitation. These bleach signals have the same formation decay kinetics (result not shown), suggesting that ligand exchange (from TOP to MUA) and solvent (from chloroform to water) does not change the quasi-type II band alignment of the CdSe/CdS QDs. However, as shown in Fig. S6,† the 1S band bleach signals of the MUA-capped water-soluble QDs recover faster compared with the TOP-capped QDs in chloroform. The kinetics can also be fitted by three exponential decay functions with a half-life of ∼1.6 to 3.1 ns (Table S2B†). The faster 1S bleach recovery is attributed to electron trapping caused by poorer surface passivation in aqueous phase and/or recombination of trapped holes (see below).^28^ PL decays in MUA-capped QDs are much faster than those in TOP-capped QDs due to efficient hole transfer to MUA ligands. This point will be further discussed later.

### Charge separation in MUA-capped CdSe/CdS QD-MV^2+^

**Fig. 4:**
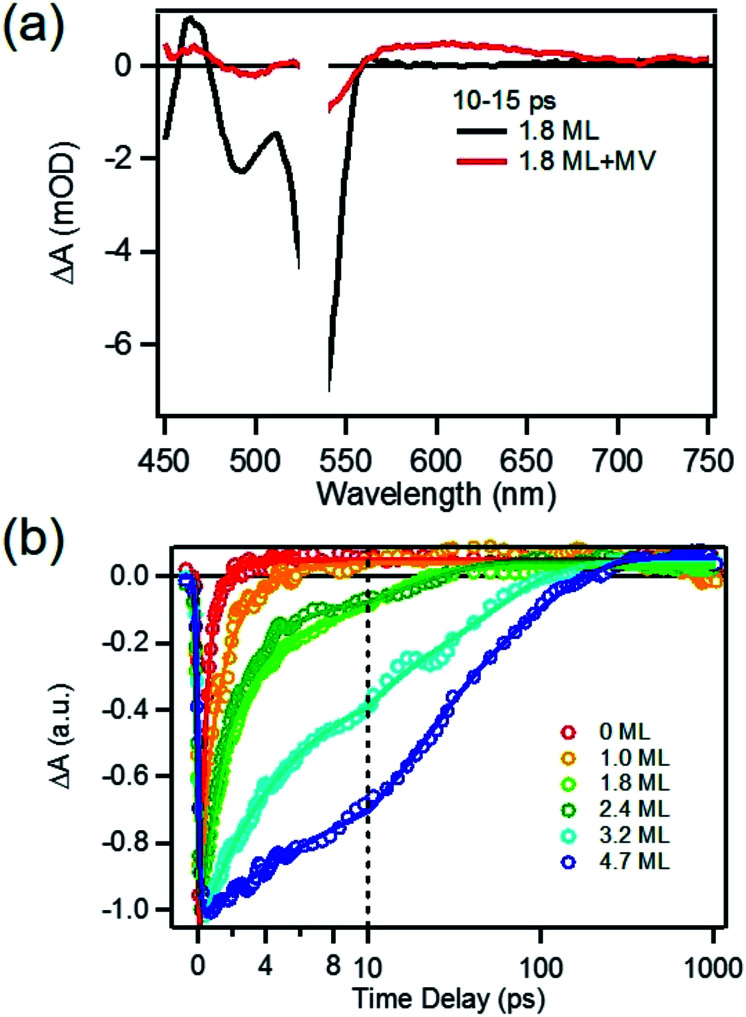
Transient spectra and kinetics of MUA-capped CdSe/CdS QD–MV^2+^ complexes in water. (a) Average TA spectra of 1.8 ML CdSe/CdS QDs with (red) and without (black) added MV^2+^ at 10–15 ps after 530 nm excitation; (b) kinetic traces of 1S exciton bleach recovery of CdSe/CdS QDs with 0, 1.0, 1.8, 2.4, 3.2 and 4.7 MLs of CdS shells QD in aqueous solution with added MV^2+^.

To determine the charge separation rate, we carried out transient absorption study of CdSe/CdS QD–MV^2+^ complexes under conditions similar to the steady-state photo-reduction measurements. The TA spectra for 1.8 ML MUA-capped CdSe/CdS QD–MV^2+^ sample were shown in Fig. 4a and the complete spectra of other QDs with different CdS shell thicknesses were shown in Fig. S7 in ESI.† Compared with free MUA capped-QDs in aqueous solution, the 1S exciton bleach undergoes much faster recovery, indicating shorter-lived 1S electrons. After complete bleach recovery, the TA spectra consist of a broad MV^+^˙ radical absorption centered at 605 nm (red line in Fig. 4a) and a Stark effect signal of exciton bands. The MV^+^˙ radical absorption spectrum agreed well with those reported in the literature^21,29^ and observed in steady-state photo-reduction experiment discussed above. The derivative like Stark effect signal, which can be clearly seen after 15 ps (when the 1S electron state filling bleach signal had nearly completely decayed), has been attributed to the shift of the exciton bands caused by the hole in the QD in the charge separated state.^21^ The formation of these spectral features and the complete recovery of 1S exciton bleach confirm the transfer of CB electrons from CdSe/CdS QDs to MV^2+^.

The 1S bleach recovery kinetics of CdSe/CdS QD–MV^2+^ as a function of CdS shell thicknesses are shown in Fig. 4b. It is clear that with increasing CdS shell thickness, the 1S bleach recovery become slower. We note that charge transfer rates scale with number of acceptors on QD surfaces.^16,35^ Because MV^2+^ is soluble in water, we measured MV^2+^ concentration dependent charge transfer kinetics to estimate the average number of MV^2+^ per QD, following a previously-reported model.^23,35^ The details of the experiments and model are provided in the ESI (Fig. S8 and ESI†), from which we estimate that the average number of MV^2+^ per QD for the data presented in Fig. 4b is ∼1 (ESI for details†). Therefore, the shell thickness dependent electron transfer rates in Fig. 4b can be directly compared. We determined the half-life of the bleach recovery to represent the effective charge separation time (*τ*_CS_), which are 0.3, 0.6, 1.5, 1.7, 6.3 and 20 ps for CdSe/CdS QDs with 0, 1.0, 1.8, 2.4, 3.2, and 4.7 MLs of shells, respectively (Tables 1 and S3†). Although the charge separation rate slows down as CdS shell thickness increasing, the ultrafast and complete bleach recovery as well as the ultrafast MV^+^˙ radical formation suggests that the initial charge separation yield is almost 100% in all CdSe/CdS QD–MV^2+^ solutions. Thus, charge separation is not the limiting step for increasing the steady-state MV^+^˙ radicals generation quantum yields in these systems.

### Charge recombination in TOP-capped CdSe/CdS QD–MV^2+^

**Fig. 5:**
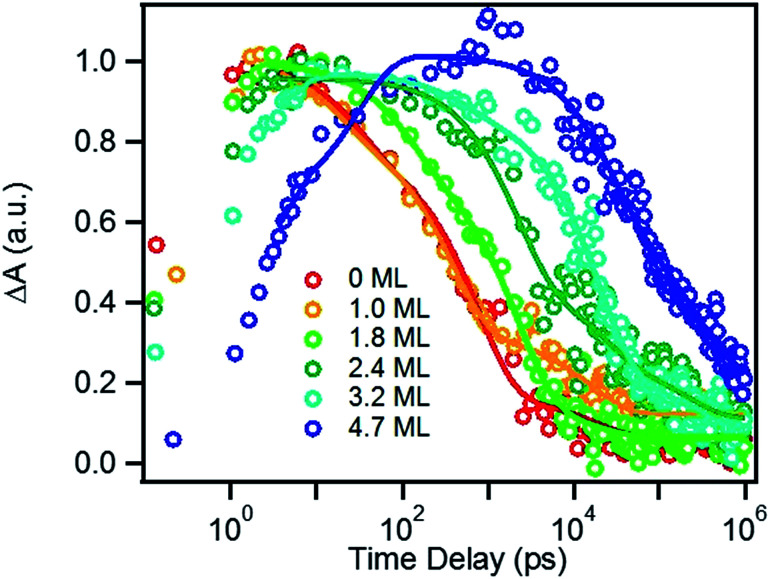
Formation and decay kinetics of MV^+^˙ radical generated by 530 nm excitation of TOP-capped CdSe/CdS QDs of varying shell thickness in chloroform.

We next investigated the relative rates of hole filling (*k*_HF_) by the sacrificial electron donor (MPA) *vs.* the charge recombination (*k*_CR_) between the hole in the QDs and the electron in MV^+^˙ radicals (Scheme 1). To measure the charge recombination rate, we investigated TOP-capped CdSe/CdS QD–MV^2+^ in chloroform in the absence of MUA and MPA (sacrificial electron donors). Under such conditions, hole removal by sacrificial donor cannot occur, and the only decay pathway for the charge separated state is the recombination of the MV^+^˙ radical with the hole in CdSe. As shown in Fig. S9† in ESI, the TA spectra measured with 530 nm excitation are similar to those observed in the aqueous solutions and the broad MV^+^˙ radical absorption band and the Stark effect signal of exciton bands can be clearly seen after 15 ps. The average kinetics of MV^+^˙ radical from 600–630 nm are compared in Fig. 5 for samples of different CdS shell thickness. The comparison shows that MV^+^˙ radicals are formed within hundreds of femtoseconds for all CdSe/CdS QDs except for the 4.7 ML CdS shell (in which the formation time is ∼100 ps). The trend is similar to that of MUA-capped CdSe/CdS QD–MV^2+^ complexes in aqueous solution. Here we focus on the radical decay kinetics, which is a measure of the charge recombination process of the electron in MV^+^˙ radicals and the hole in the QDs. These kinetics were well fit by multiexponential functions (Fig. S7†), from which the half-life times were determined (Table 1) to approximately represent the charge recombination time (*τ*_CR_). The charge recombination times increase down systematically from 0.5 ns for the CdSe core to 100 ns for the CdSe/CdS QD with 4.7 ML CdS shell.

### Hole transfer in MUA-capped CdSe/CdS

Previous studies have shown that hole removal is also critical to the suppression of charge recombination^21,28,36,37^ and hole transfer rates to sacrificial electron donors can be measured by time-resolved photoluminescence (PL) decay of CdSe/CdS QDs in the presence of electron donors.^21,28^ In this study, MUA is not only the native ligand but also an efficient hole acceptor (or electron donor). To measure the hole-transfer rate, we compared the PL decay rates of MUA-capped CdSe/CdS QDs in aqueous solutions and TOP-capped QDs in chloroform under similar experimental conditions (excitation power and sample absorbance) as shown in Fig. S10.† Compared to TOP-capped QDs, MUA-capped QDs show a much faster PL decay, indicating effective hole transfer to the MUA ligand. To quantify the hole-transfer rates, these kinetics were fitted to multi-exponential decay functions (Table S7†). We determined the decay rates from the half-life times obtained from the fit for TOP-capped (*k*_QD_) and MUA-capped (*k*_QD-MUA_) QDs. PL decay in MUA-capped QDs is determined by hole transfer to MUA (*k*_HT_), electron trapping induced by MUA ligand exchange (*k*_1S-MUA_) and intrinsic electron and hole radiative and nonradiative decay (*k*_QD_) in native TOP-capped QDs, *i.e. k*_QD-MUA_ = *k*_HT_ + *k*_1S-MUA_ + *k*_QD_. The effect of MUA ligand exchange on 1S electron lifetime (*k*_1S-MUA_) has been measured in Fig. 3 and S6.† Finally, the hole transfer rates to MUA were calculated according to eqn (2) below:^28,35^

2 *k*_HT_ = *k*_QD-MUA_ − *k*_QD_ − *k*_1S-MUA_

The calculated average hole transfer lifetimes (*τ*_HT_ = 1/*k*_HT_) are listed in Table 1.

### Shell thickness dependent electron transfer, charge recombination and hole transfer rates

**Fig. 6:**
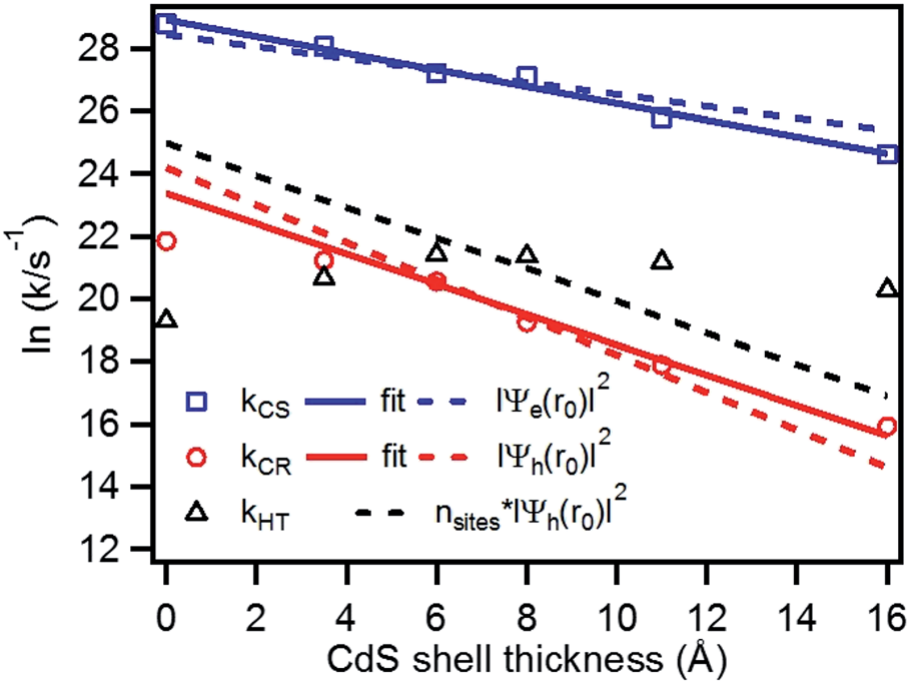
Plot of the logarithm of charge separation (blue square), charge recombination (red circle) and hole transfer rates (black triangle) as a function of the CdS shell thickness. Also shown are fits to the measured electron (solid blue line) and hole (solid red line) rates according to eqn 3, the calculated electron (blue dashed line) and hole (red dashed line) densities at the shell surface, as well as the product of hole densities and the number of surface Cd atoms as a function of the CdS shell thickness.

We have plotted the logarithm of the charge separation, recombination and hole transfer rates as a function of CdS shell thickness as shown in Fig. 6. It shows that both the charge separation and recombination rates decay exponentially with the CdS shell thickness. These decays can be fit by eqn (3).

3 *k*(*d*) = *k*_0_e^−*βd*^

here, *k*_0_ is the charge separation or recombination rate for free QDs, *β* is the decay constant and *d* is the CdS shell thickness in Å. Best fit of the thickness dependence yields a slope *β* of 0.26 ± 0.03 and 0.48 ± 0.06 Å^−1^ for charge separation and recombination rates, respectively. It is interesting to note that these exponential decay constants are much smaller than the values reported for CdSe/ZnS type I core/shell QDs (0.35 ± 0.03 and 0.91 ± 0.14 Å^−1^ for charge separation and recombination, respectively).^12^

It has been demonstrated that the rate electron transfer from an excited QD can be described by Auger-assisted electron transfer model:^24^4
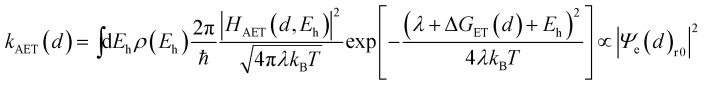
In eqn (4), where Δ*G*_ET_(*d*) is the free energy difference between electron in the CB of QD and in the acceptor, *E*_h_ is the energy of the excited hole level involved in the Auger assisted ET process, *H* the electronic coupling strength, and *λ* the total reorganization energy.

The rate of charge recombination processes is given by eqn (5):5
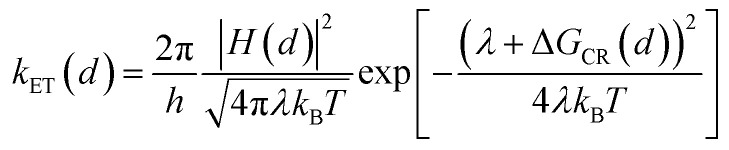
where Δ*G*_CR_, and *H* are the free energy change and electronic coupling strength for charge recombination.

To quantify the effect of CdS shell thickness on the electron (hole) transfer rate, we calculate the Eigen function and energy of the electrons and holes in CdSe/CdS QDs by modelling them as particles confined in spherical wells of finite depth (see detail in ESI†). Fig. S12† shows the radial distribution functions of the 1S electron and hole in CdSe core as well as QDs with different CdS shells. It is clear that the electron and hole wave functions spread into the CdS layer, but their amplitudes at the CdS surface decay drastically with the increasing shell thickness. The electronic coupling strength for charge separation and recombination should depend on the overlap integrals of the electron (or hole) wave function with the LUMO of MV^2+^ and can be assumed to be proportional to the amplitude of the electron (or hole) wave function *Ψ*_e_(*d*)_r0_(*Ψ*_h_(*d*)_r0_) at the QD/ligand interface. As shown in Fig. 6, the calculated surface electron and hole densities decay exponentially with CdS layer thickness with exponential decay factors of 0.19 Å^−1^ for the electron and 0.59 Å^−1^ for the hole. In this plot, we have scaled the electron (hole) density to the values on the CdSe core to match the charge separation (recombination) rate. The trend of electron and hole densities change with shell thickness agrees reasonably well with the measured dependence of charge separation and recombination rates, respectively. A similar exponential dependence of ET rate with the thickness of the insulating spacer has been extensively studied in QD-molecular systems and in dye-sensitized oxide nanoparticles.^12,16,38–40^ This results suggests that, in CdSe/CdS QDs, the CdS shell serves as a tunnelling barrier for the electron and hole transfer, and it slows down the rates by decreasing the electronic coupling with the adsorbate MV^2+^.

As shown in eqn (4) and (5), in addition to the electronic coupling strength, charge separation and recombination rates also depend on the reorganization energy and driving force. The good agreement discussed above suggests that these factors play a minor role in determining the shell thickness dependence of charge separation and recombination rates in these systems. The ET reorganization energy contains negligible contribution from CdSe/CdS QDs because of the delocalized 1S wave function, and is controlled by that of the electron acceptor (MV^2+^). This value (estimated to be ∼0.3 eV (ref. 24)) should be independent of the CdS shell thicknesses. The driving force for charge separation, Δ*G*_ET_(*d*), decreases by ∼300 meV from CdSe core to 4.7 ML CdSe/CdS QDs, which should also contribute to additional decrease of ET rate with shell thickness and may explain the slight difference between the measured *β* value of (0.26 ± 0.03 Å^−1^) and that computed from coupling strength change alone (0.19 Å^−1^). This is consistent with the finding of a previous study which have shown that Auger assisted ET rates increases slowly with driving force at −Δ*G*_ET_(*d*) > *λ*, which is driving force region of CdSe/CdS–MV^2+^ complexes.^24^ For the charge recombination process, the driving force should change negligibly on the shell thickness because the energy of VB hole only depends weakly on shell thickness in quasi-type II CdSe/CdS QDs (Scheme 1).

Interestingly, Fig. 6 shows that there is no systematic CdS shell thickness dependence in the hole transfer lifetimes from the QD to MUA ligands: the hole transfer times are relatively slow (4.2 ns) in the CdSe core-only QDs and are similar for all core/shell QDs with 1 to 4.7 ML of CdS shell (see Table 1). The coupling strength of hole transfer should scales with hole density at the QD surface with a *β* value of 0.59 Å^−1^. However, unlike charge recombination between one MV^+^˙ radical, for which there is only one hole–radical pair per QD, hole transfer rate to the sacrificial electron donors should be proportional to the number of MUA ligands on the QD surface, which should scale with the surface area. The number of MUA ligand binding sites on the QD surface is often assumed to be proportional to the surface area.^23^ In Fig. 6, we show a plot of the product of surface hole density and number of MUA ligands binding sites on QD surface as a function of shell thickness. The trend of this product does not agree with the measured dependence of hole transfer rate. This result may suggest that the number of MUA ligands on the QD surface increase much faster with the shell thickness than the trend expected from the surface area. We hypothesize^41,42^ that with increasing shell thickness, the QD surface curvature decreases, which increases the packing density of the MUA ligand layers.^41,42^ As a result, the reduction of hole density at QD surface is compensated by an increase of MUA ligand density on larger QDs, resulting in the negligible shell-thickness dependence of the observed hole transfer rates.

### Origin of shell thickness dependent steady-state photon reduction quantum efficiency

As shown in Scheme 1b, the overall efficiency of photoreduction depends on the competition of forward and backward charge transfer processes. In all CdSe/CdS QDs-MV^2+^ systems studied the initial charge separation quantum efficiencies are ∼100% because the charge separation rates (Fig. 4) are much faster than the intrinsic electron–hole recombination rates in all CdSe/CdS QDs (Fig. 3 and S6†). On the other hand, the charge recombination times (*τ*_CR_) increases with while the hole transfer times depend weakly on the CdS shell thickness. As shown in Fig. 6, for CdSe/CdS shell with thin shells, the charge recombination time is faster than the hole transfer time, which gives rise to charge recombination loss. Compared to CdSe core only QDs, the charge separation rate in 4.7 ML CdSe/CdS QDs is reduced by 200 times, which is ∼70 times faster than the hole transfer time, reducing the charge recombination loss. Thus, the enhancement of the steady state photoreduction quantum yield in core/shell QDs can be attributed the unique morphology of quasi-type II core/shell QDs, which leads to an exponential decrease of charge recombination rate with shell thickness and much weaker change of hole transfer rates to the sacrificial electron donors. Similar trend can also be expected in type II core/shell QDs.

## Conclusions

In summary, we have studied MV^2+^ photo-reduction using quasi-type II CdSe/CdS core/shell QDs as a function of CdS shell thicknesses. We observed a systematic increase of MV^+^˙ radical photogeneration quantum yield with the CdS shell thickness from 12.7(±1.2)% in CdSe core only QDs to 26.5(±1.7)% in CdSe/CdS QDs with 4.7 ML of CdS. We have investigated how the shell thickness affects the charge separation, charge recombination and hole transfer rates in these CdSe/CdS QDs by time-resolved transient absorption and fluorescence decay spectroscopy. With increasing CdS shell thickness, both the rates of charge separation and recombination decreased exponentially with exponential decay factors of 0.26 ± 0.03 and 0.48 ± 0.06 Å^−1^, respectively. Model calculations of these core/shell QDs showed that the trends of charge separation and recombination rates agreed with the exponential decreases of the electron and hole densities at the QD surface with the shell thickness. The hole transfer rates were nearly independent of the shell thickness, which was tentatively attributed to an increase of surface ligand (also the electron donor) density at larger QDs. In all QDs, the charge separation times is faster than the lifetime of CB electrons in free QDs, leading to ∼100% initial photo-reduction. With increasing shell thickness, the recombination rate is retarded exponentially to reduce the recombination loss, enhancing the steady state quantum efficiencies of MV^2+^ photoreduction. The interplay of different thickness dependent electron and hole transfer coupling strength and the surface area dependent adsorbed sacrificial electron donor numbers gives rise to an interesting and likely general scheme for optimizing photoreduction quantum yield by controlling the thickness and nature of the shell materials in quasi-type II and type II core/shell QDs.

## Supplementary Material

SC-007-C6SC00192K-s001
