# Supplementary material for: Enhancing photo-reduction quantum efficiency using quasi-type II core/shell quantum dots
Source: Chem Sci. 2016 Mar 2;7(7):4125–33. doi: 10.1039/c6sc00192k (PMC6013914; doi:10.1039/c6sc00192k)
Supplement: SC-007-C6SC00192K-s001 [file SC-007-C6SC00192K-s001.pdf]

Electronic Supplementary Material (ESI) for Chemical Science.
This journal is © The Royal Society of Chemistry 2016

Supporting Information for:

Enhancing Photo-reduction Quantum Efficiency Using Quasi-Type II Core/Shell Quantum Dots

Yanyan Jia[1,2]†, Jinquan Chen[1]†, Kaifeng Wu[1], Alexey L. Kaledin[2], Djamaladdin G. Musaev[2], Zhaoxiong Xie[3] and Tianquan Lian[1]

[1]Department of Chemistry, Emory University, Atlanta, Georgia 30322, United States

[2]Cherry L. Emerson Center for Scientific Computation, Emory University, Atlanta, Georgia 30322, United States

[3]State Key Laboratory of Physical Chemistry of Solid Surfaces and Department of Chemistry, College of Chemistry and Chemical Engineering, Xiamen University, Xiamen 361005, China

Supporting Information List:

SI 1. Sample preparation and Characterization

SI 2. Pump-probe Transient Absorption Spectroscopy Setup

SI 3. Steady state difference UV-Vis spectra of MV^{2+} photo-reduction using different CdSe/CdS core/shell QDs.

SI 4. Full TA spectra of free CdSe/CdS QDs in chloroform.

SI 5. Kinetic traces of B1, B2 and B3 of free TOP-capped CdSe/CdS QDs in chloroform.

SI 6. Full TA spectra of free CdSe/CdS QDs in aqueous solutions.

SI 7. 1S exciton bleach kinetics of free CdSe/CdS QDs in aqueous solutions.

SI 8. TA spectra of CdSe/CdS-MV^{2+} in aqueous solutions.

SI 9. 1S exciton bleach kinetic trace of CdSe/CdS QDs with 0, 1.8 and 4.7 ML CdS shell with different concentration of MV^{2+} in solutions.

SI 10. TA spectra of CdSe/CdS-MV^{2+} in chloroform.

SI 11. Comparison of time-resolved photoluminescence (PL) decay between MUA-capped CdSe/CdS QDs in aqueous solutions and TOP-capped QDs in chloroform.

SI 12. Comparison of time-resolved PL decay and 1S bleach TA signals of TOP-capped QDs in chloroform.

SI 13. Radial distribution function of 1S conduction-band electron and valence-band hole of quasi-type II CdSe/CdS quantum dots.

SI 14. Fitting parameters of 1S exciton band in free CdSe/CdS QDs in chloroform and aqueous solutions.

SI 15. Fitting parameters of electron transfer rate of CdSe/CdS-MV^{2+} 1S exciton band kinetics.

SI 16. Fitting parameters of average number (*m*) of adsorbed MV^{2+} molecules for 1.8 and 4.7 ML CdSe/CdS QDs.

SI 17. Fitting parameters of Langmuir isotherm to the mean surface coverage and MV^{2+} concentration.

SI 18. Fitting parameters of MV^{2+} formation and decay kinetics.

SI 19. Fitting parameters of time-resolved photoluminescence (PL) decay of CdSe/CdS QDs in aqueous solutions and in chloroform.

SI 20. Theoretical methods.

SI 21. Reference.

SI 1. Sample preparation and Characterization

Chemicals:

Cadmium oxide (CdO, 99.998%), octadecylphosphonic acid (ODPA, 97%), trioctylphosphine oxide (TOPO 99%), trioctylphosphine (TOP, 97%), selenium powder (99.999%), sulphur powder (99.999%), oleic acid (OA, 90%), 1-octadecene (ODE, 90%), ethanol (99%) and chloroform (99%) were purchased from Sigma Aldrich. All chemicals were used without further purification.

Synthesis of CdSe quantum dots: CdSe cores were synthesized according to a literature method.[1] Briefly, 60 mg CdO, 280 mg ODPA and 3 g TOPO were added to a 25 mL flask. The mixture was degassed under vacuum for 30 min and heated to 150 °C. And then the reaction mixture was heated to 320 °C to form a colorless clear solution under nitrogen flow. At this point, 1.0 mL TOP was injected to the solution, and the temperature was brought up to 380 °C, at which point Se/TOP solution (60 mg Se in 0.5 mL TOP) was swiftly injected into the flask. When the CdSe core nanocrystals reached the desired size, the reaction was terminated by removing the heat. The as-prepared CdSe nanoparticles were precipitated by adding ethanol and dispersed in chloroform as a stock solution.

Synthesis of CdSe/CdS core/shell nanostructures: The CdS shell was grown by successive ion layer adsorption and reaction (SILAR) method.[2] A 0.1 M cadmium precursor solution was prepared by dissolving 0.064 g of CdO in 2.0 mL of OA and 7.5 mL of ODE at 300 °C to obtain a colorless solution. The precursor solution was then maintained at room temperature. A 0.1 M sulfur injection solution was prepared by dissolving 32 mg of sulfur in 10 mL of ODE in an ultrasonic bath. For the CdS shell growth reaction, a chloroform solution containing 100 nmol of CdSe QDs was loaded in

a mixture of ODE (5 mL). The reaction solution was degassed under vacuum at room temperature for 1 hour and 150 °C for 30 min to completely remove the chloroform, water and oxygen inside the reaction solution. After that the reaction solution was heated up to 200 ^{o}C, a desired amount of cadmium precursor and sulfur precursor mixed solution were injected dropwise into the growth solution at a rate of 1 mL/hr using a syringe pump. After finishing precursor infusion, the reaction solution was heated up to 230 ^{o}C and remained for 30 min. The producing CdSe/CdS core/shell nanoparticles were precipitated by adding ethanol and dispersed in chloroform as a stock solution.

The CdS shell monolayer calculation: The spherical concentric shell model (CSM) was employed to calculate the amount of shell precursor necessary for the growth of each monolayer. Hereafter, referral to a monolayer (ML) will be taken to mean a thickness equal to half the c-lattice parameter of the bulk semiconductor, 0.34 nm in the case of CdS.[3]

Table S1. CdSe/CdS core/shell nanoparticles size and CdS shell thickness

Sample	Size (d/nm)	CdS shell thickness (nm)	CdS shell thickness
CdSe core	2.8		0 ML
CdSe/CdS #1	3.5	0.35	1.0 ML
CdSe/CdS #2	4.0	0.60	1.8 ML
CdSe/CdS #3	4.4	0.80	2.4 ML
CdSe/CdS #4	5.0	1.10	3.2 ML
CdSe/CdS #5	6.0	1.60	4.7 ML

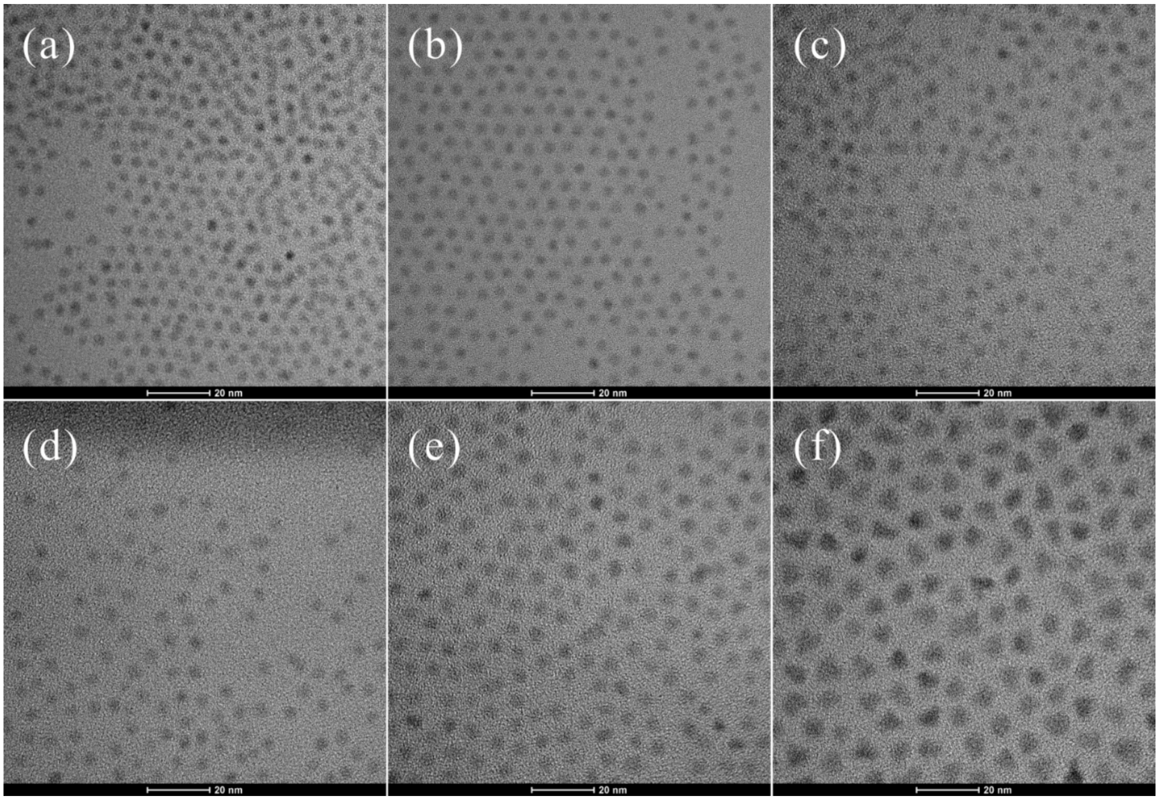

Figure S1. TEM images of CdSe seeds and CdSe/CdS core/shell nanostrucures with different shell thickness

Ligand exchange to make water soluble QDs: The CdSe QDs prepared above were transformed into water by ligand exchange with 11-mercaptoundecanoic acid (MUA). Excess amount of MUA (30 μg) was dissolved in 10 mL H_2O and the pH of the solution was adjusted to above 14 with sodium hydroxide. A few ml CdSe QD solution were added and the solution was stirred overnight. The MUA capped QDs were precipitated with methanol and isolated by centrifugation and decantation. After drying, the precipitate was re-dissolved in H_2O for further use.

SI 2. Pump-probe Transient Absorption Spectroscopy Setup

Femtosecond Transient Absorption (TA). The transient absorption measurements were conducted in a Helios spectrometer (Ultrafast Systems LLC) with pump and probe beams derived from a regenerative amplified Ti:Sapphire laser system (Coherent Legend, 800 nm, 150 fs, 3 mJ/pulse, and 1 kHz repetition rate). 50% of the 800 nm output pulse was used to pump an Optical Parametric Amplifier (Opera, Coherent) to generate two tunable near-IR pulses, signal and idler, from 1.1 to 2.5 um. Signal and idler beams were separated with a dichroic mirror, and the former was used to generate 530 nm excitation beams by mixing with another small portion (~7%) of the 800 nm output pulse in a BBO crystal. A series of neutral-density filter wheels were used to adjust the power the pump beam. The pump beam was focused at the sample with a beam waist of about 300 μm. A white light continuum (WLC) from 420 to 800 nm was generated by attenuating and focusing ∼10 μJ of the 800 nm pulse into a sapphire window. The WLC was split into a probe and reference beam. The probe beam was focused with an Al parabolic reflector onto the sample (with a beam waist of 150 μm at the sample). The reference and probe beams were focused into a fiber optics-coupled multichannel spectrometer with complementary metal-oxide-semiconductor (CMOS) sensors and detected at a frequency of 1 kHz. The intensities of the referencep and probe beams were ratioed to correct for pulse-to-pulse fluctuation of the white-light continuum. The delay between the pump and probe pulses was controlled by a motorized delay stage. The pump beam was chopped by a synchronized chopper to 500 Hz. The change in absorbance for the pumped and unpumped samples was calculated. 1 mm cuvettes were used for all spectroscopy measurements. The instrument response function (IRF) of this system was measured to

be ~150 fs by measuring solvent responses under the same experimental conditions (with the exception of a higher excitation power).

Nanosecond Transient Absorption. Nanosecond TA was performed with the EOS spectrometer (Ultrafast Systems LLC). The pump beam at 400 nm was generated in the same way as femtosecond TA experiments. The white light continuum (380-1700 nm, 0.5 ns pulse width, 20 kHz repetition rate) used here was generated by focusing a Nd:YAG laser into a photonic crystal fiber. The delay time between the pump and probe beam was controlled by a digital delay generator (CNT-90, Pendulum Instruments). The probe and reference beams were detected with the same multichannel spectrometers used in femtosecond TA experiments. The IRF of this system was measured to be ~280 ps.

SI 3. Steady state difference UV-Vis spectra of MV^{2+} photo-reduction using different CdSe/CdS core/shell QDs.

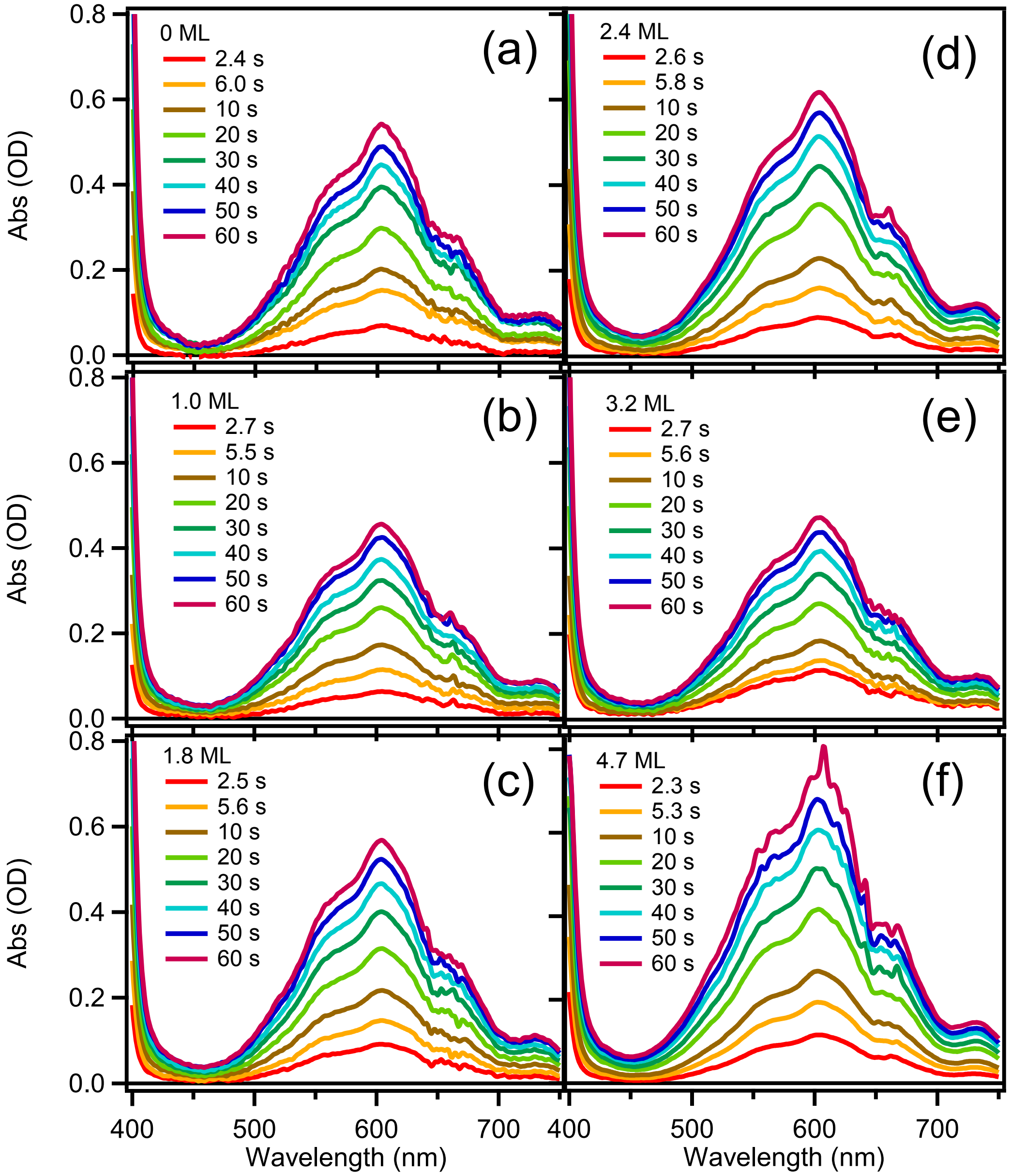

Figure S2. UV−vis difference spectra (after−before irradiation) of solutions containing CdSe/CdS QDs with (a) 0, (b) 1.0, (c) 1.8, (d) 2.4, (e) 3.2, and (f) 4.7 MLs CdS shell with 5 mM MV^{2+} and 50 mM MPA under 405 nm (23.8 mW) illumination.

SI 4. Full TA spectra of free TOP-capped CdSe/CdS QDs in chloroform.

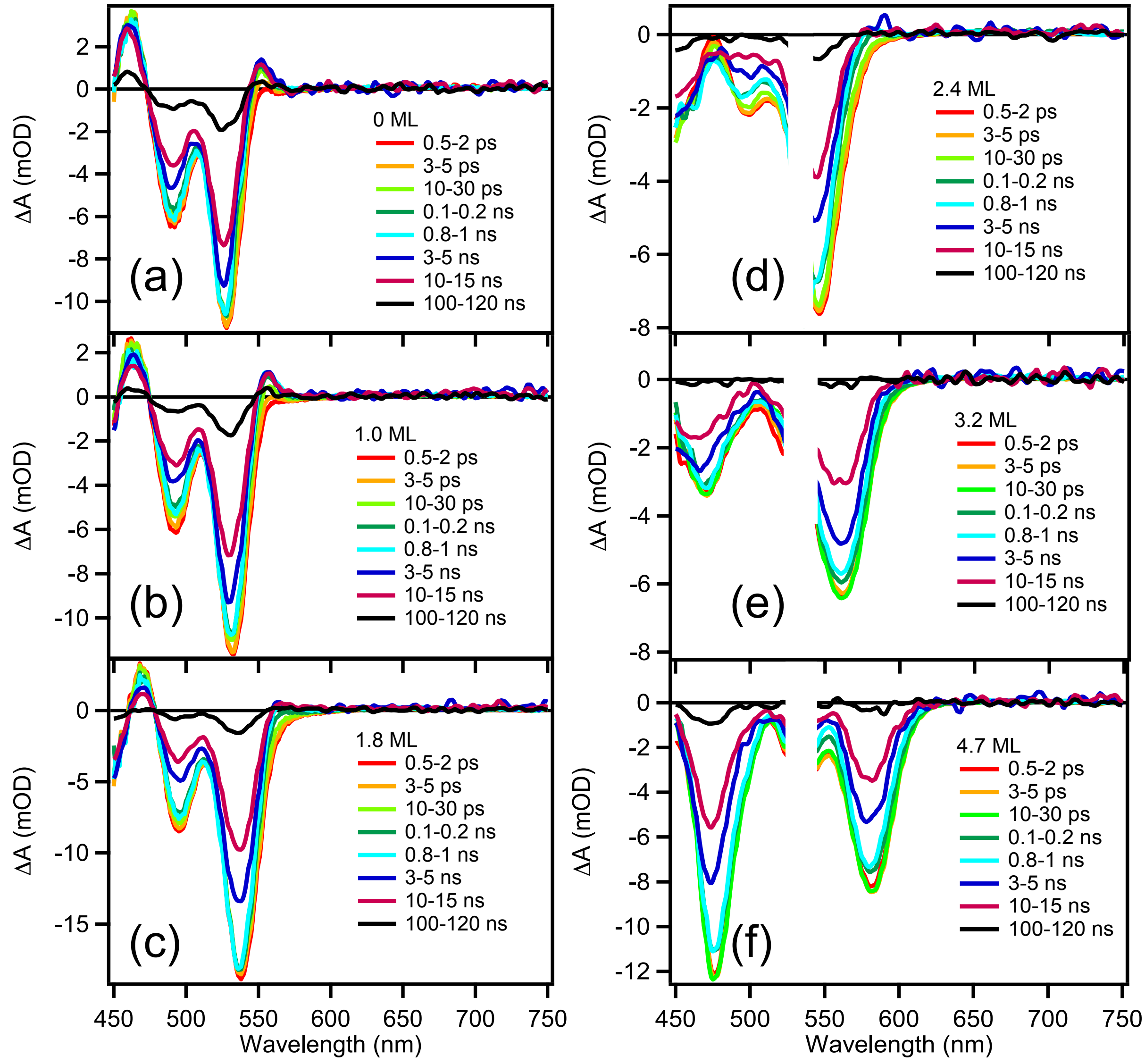

Figure S3. Transient absorption spectra of TOP-capped CdSe/CdS QDs with (a) 0, (b) 1.0, (c) 1.8, (d) 2.4, (e) 3.2, and (f) 4.7 MLs of CdS shell in chloroform at indicated delay times after 530 nm excitation.

SI 5. Kinetic traces of B1, B2 and B3 of free TOP-capped CdSe/CdS QDs in chloroform.

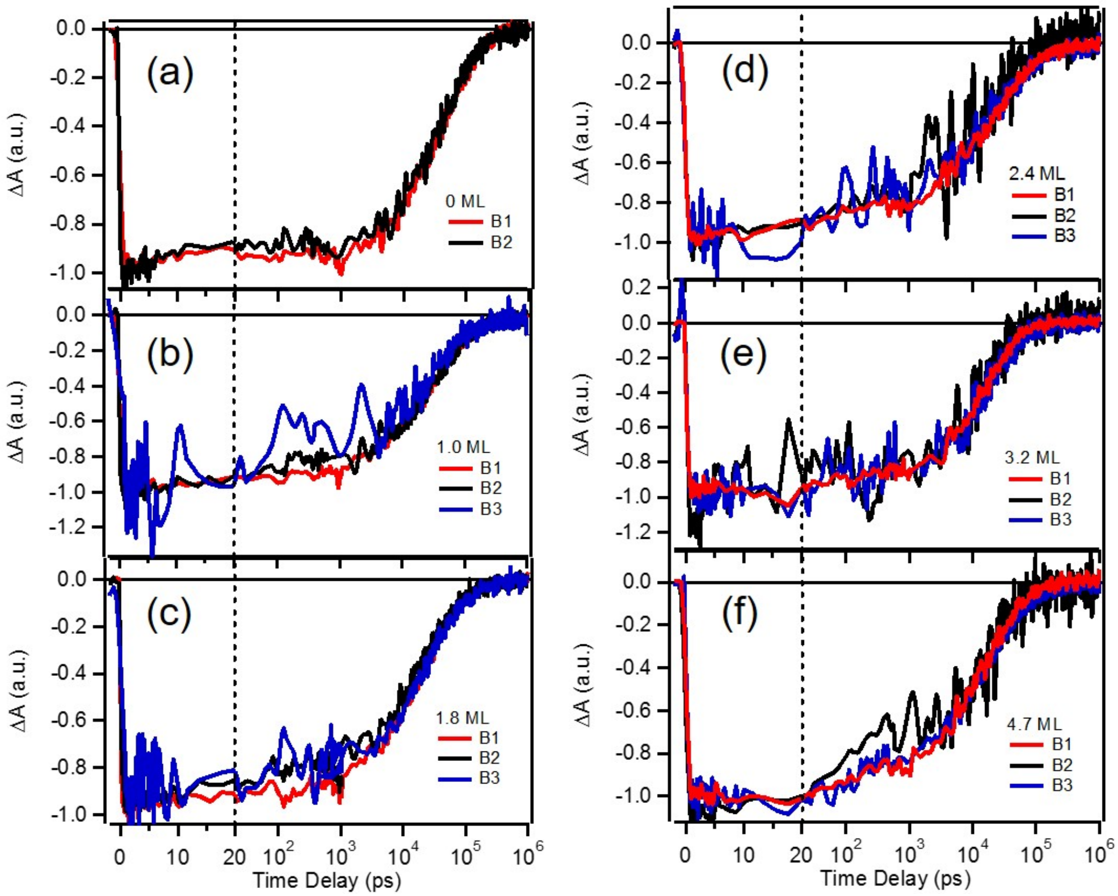

Figure S4. Comparison between B1, B2 and B3 blech kinetics of TOP-capped CdSe/CdS QDs with (a) 0, (b) 1.0, (c) 1.8, (d) 2.4, (e) 3.2, and (f) 4.7 MLs of CdS shell in chloroform after 530 nm excitation.

SI 6. Full TA spectra of free MUA-capped CdSe/CdS QDs in aqueous solutions.

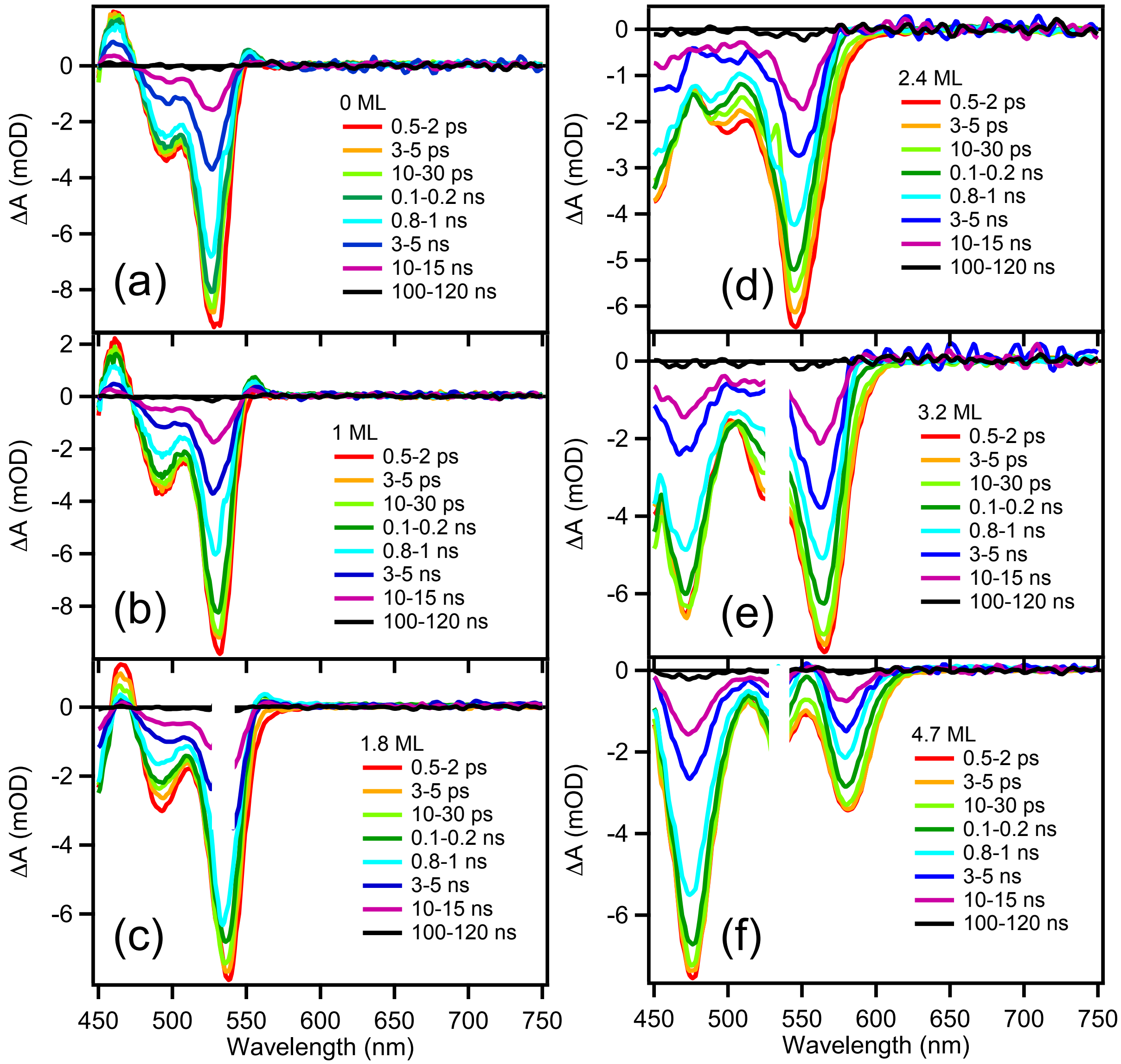

Figure S5. Transient absorption spectra of MUA-capped CdSe/CdS QDs with (a) 0, (b) 1.0, (c) 1.8, (d) 2.4, (e) 3.2, and (f) 4.7 MLs of CdS shell in aqueous solutions at indicated delay times after 530 nm excitation.

SI 7. 1S exciton bleach kinetics of free MUA-capped CdSe/CdS QDs in aqueous solutions.

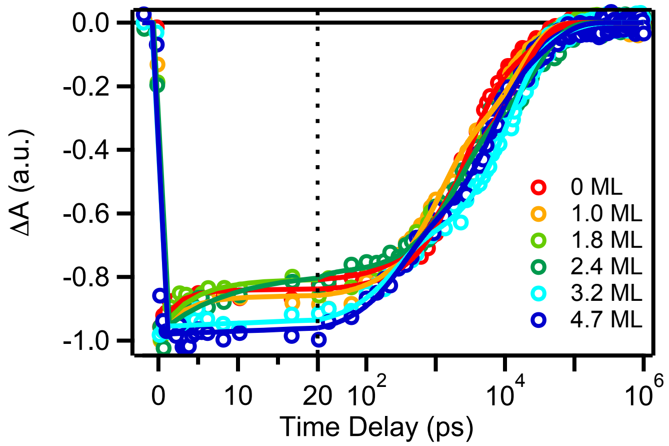

Figure S6. Comparison of 1S exciton bleach recovery kinetics in CdSe QDs with 0, 1.0, 1.8, 2.4, 3.2 and 4.7 MLs of CdS shells in aqueous solutions

SI 8. TA spectra of MUA-capped CdSe/CdS-MV^{2+} in aqueous solutions.

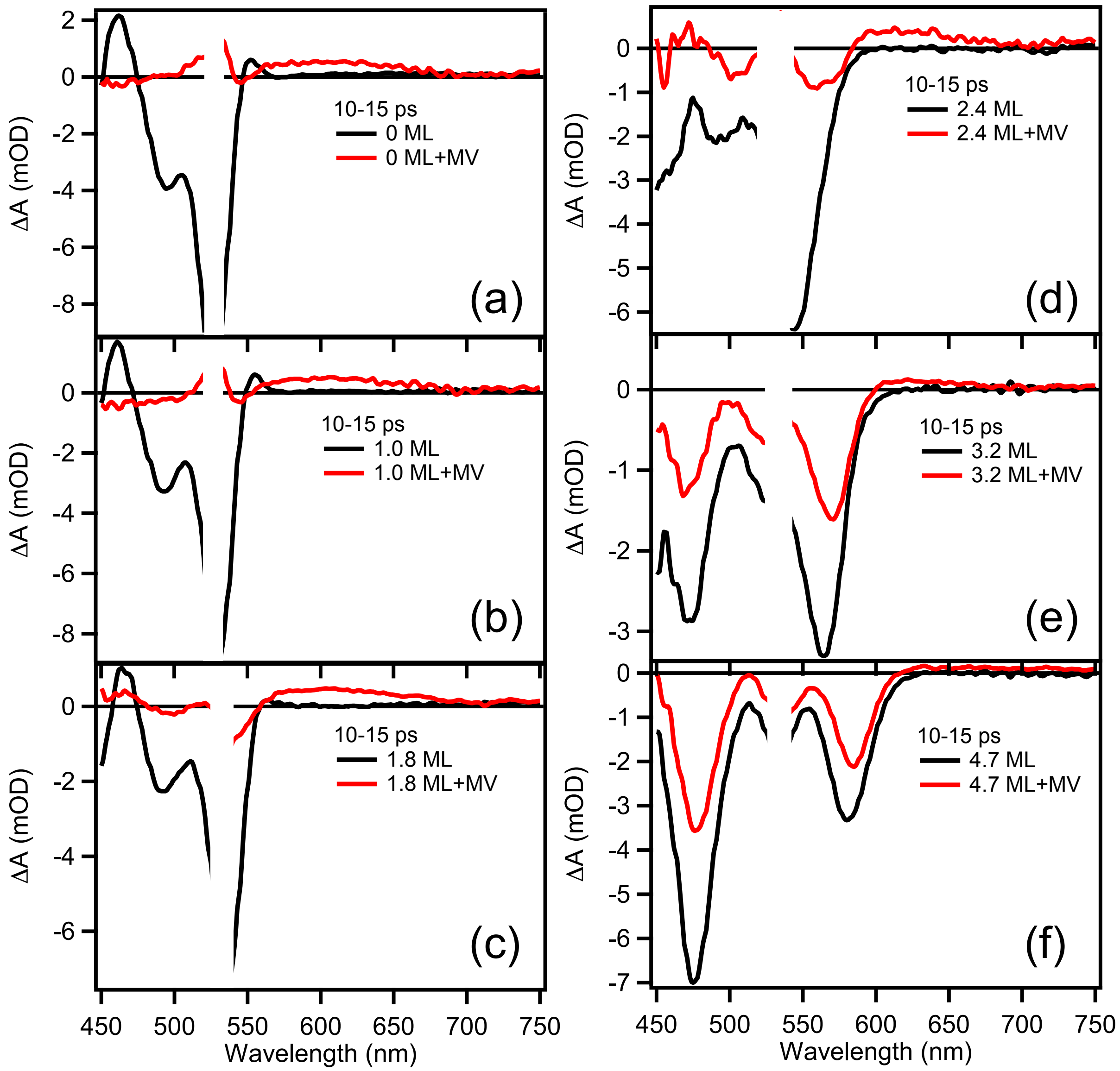

Figure S7. Transient absorption spectra of CdSe/CdS QDs with (a) 0, (b) 1.0, (c) 1.8, (d) 2.4, (e) 3.2, and (f) 4.7 MLs of CdS shell with and without MV^{2+} in aqueous solutions at indicated delay times after 530 nm excitation.

SI 9. 1S exciton bleach kinetic trace of CdSe/CdS QDs with 0, 1.8 and 4.7 ML CdS shell with different concentration of MV^{2+} in solutions.

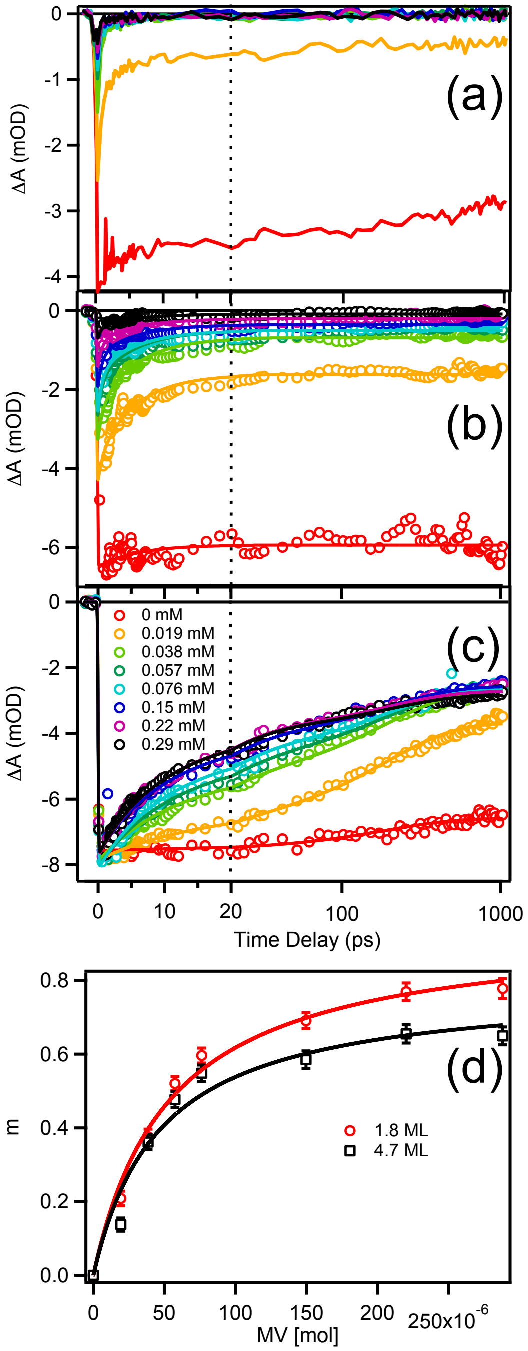

Figure S8. 1S bleach kinetic traces of CdSe with (a) 0 ML, (b) 1.8 MLs and (c) 4.7 MLs CdS shell with indicated MV^{2+} concentration; and (d) the average number (*m*) of

adsorbed MV^{2+} on CdSe/CdS QDs as a function of free MV^{2+} in solution (solid line is the Langmuir fit).

Method to determine the average number of adsorbed MV^{2+} (m) on CdSe/CdS quantum dots with different sizes: Pervious studies on single QD[4-6] and ensemble-averaged QD solutions[7-9] have shown that adsorption of acceptors on QD surfaces follow a Poisson distribution. The probability of finding QDs with n adsorbed molecules (MV^{2+} is this study) is

$$P(n,m) = (\frac{m^n e^{-m}}{n!}) \qquad \text{(Eq. S1)}$$

where m is the average number of adsorbed molecules. The ensemble averaged 1S exciton bleach recovery kinetics is given by:

$$N(t,m) = N(0)(\sum_n e^{-nk_{int}t}P(n,m))S_{free}(t) = N(0)(e^{m[e^{-k_{int}t}-1]})S_{free}(t) \quad \text{(Eq. S2)}$$

where k_{int} is the intrinsic electron transfer rate constant between a QD and an adsorbed MV^{2+}, N(t,m) and N(0) are population at time t and 0, and $S_{\text{free}}(t)$ is the bleach recovery kinetics of free QDs (without MV^{2+}). $S_{\text{free}}(t)$ was independently determined by fitting the free QD kinetics by one exponential decay function with an offset (Table S4A). Equation S2 was then used to globally fit the adsorbate concentration dependent kinetics (Figure S7 (a), (b) and (c)) with k_{int} and m as the only fitting parameters. The obtained k_{int} and m values are listed in Table S4B.

The m values obtained from the fit of MV^{2+} concentration dependent kinetics are plotted as a function of total MV^{2+} concentration in solution in Figure S7 (d). The adsorbed number of MV^{2+} on QD surface should follow the Langmuir adsorption isotherm Equation S3.[9]

$$\theta = \frac{m}{A_0} = \frac{K_0[MV^{2+}]}{1+K_0[MV^{2+}]} \qquad \text{(Eq. S3)}$$

Here θ is the mean fractional surface coverage of MV^{2+} on QDs, A_0 is the total number of available adsorption sites per QD, K_0 is the binding constant of MV^{2+}. Figure S7 (d) was fit by Equation S3, from which we obtained A_0 and K_0 (see Table S5). The fits revealed the available adsorption site (A_0) values were 0.9 ± 0.1 and 0.8 ± 0.1 for 1.8 ML and 4.7 ML CdSe/CdS QDs, respectively. (Table S5)

SI 10. TA spectra of TOP-capped CdSe/CdS-MV^{2+} in chloroform.

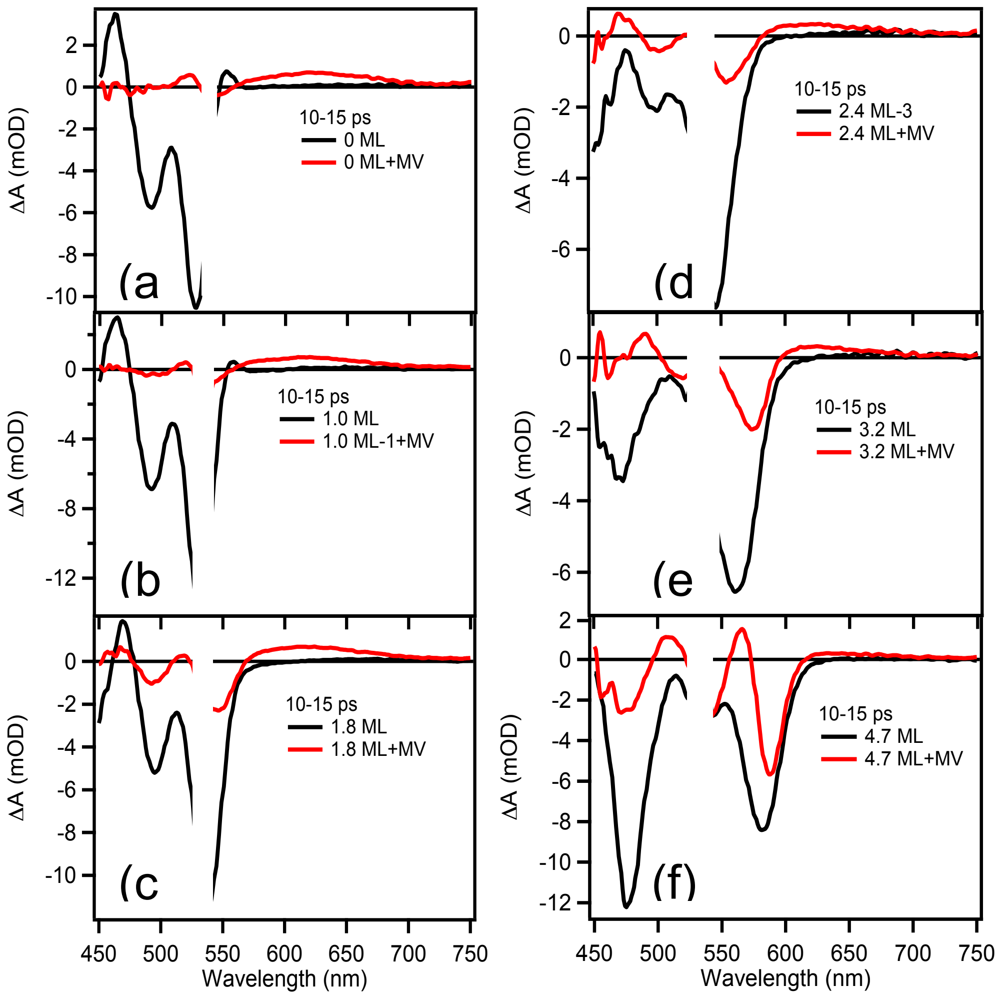

Figure S9. Transient absorption spectra of TOP-capped CdSe/CdS QDs with (a) 0, (b) 1.0, (c) 1.8, (d) 2.4, (e) 3.2, and (f) 4.7 MLs of CdS shell in chloroform with (red curve) and without (black curve) MV^{2+} at indicated delay times after 530 nm excitation.

SI 11. Comparison of time-resolved photoluminescence (PL) decay between MUA-capped CdSe/CdS QDs in aqueous solutions and TOP-capped QDs in chloroform.

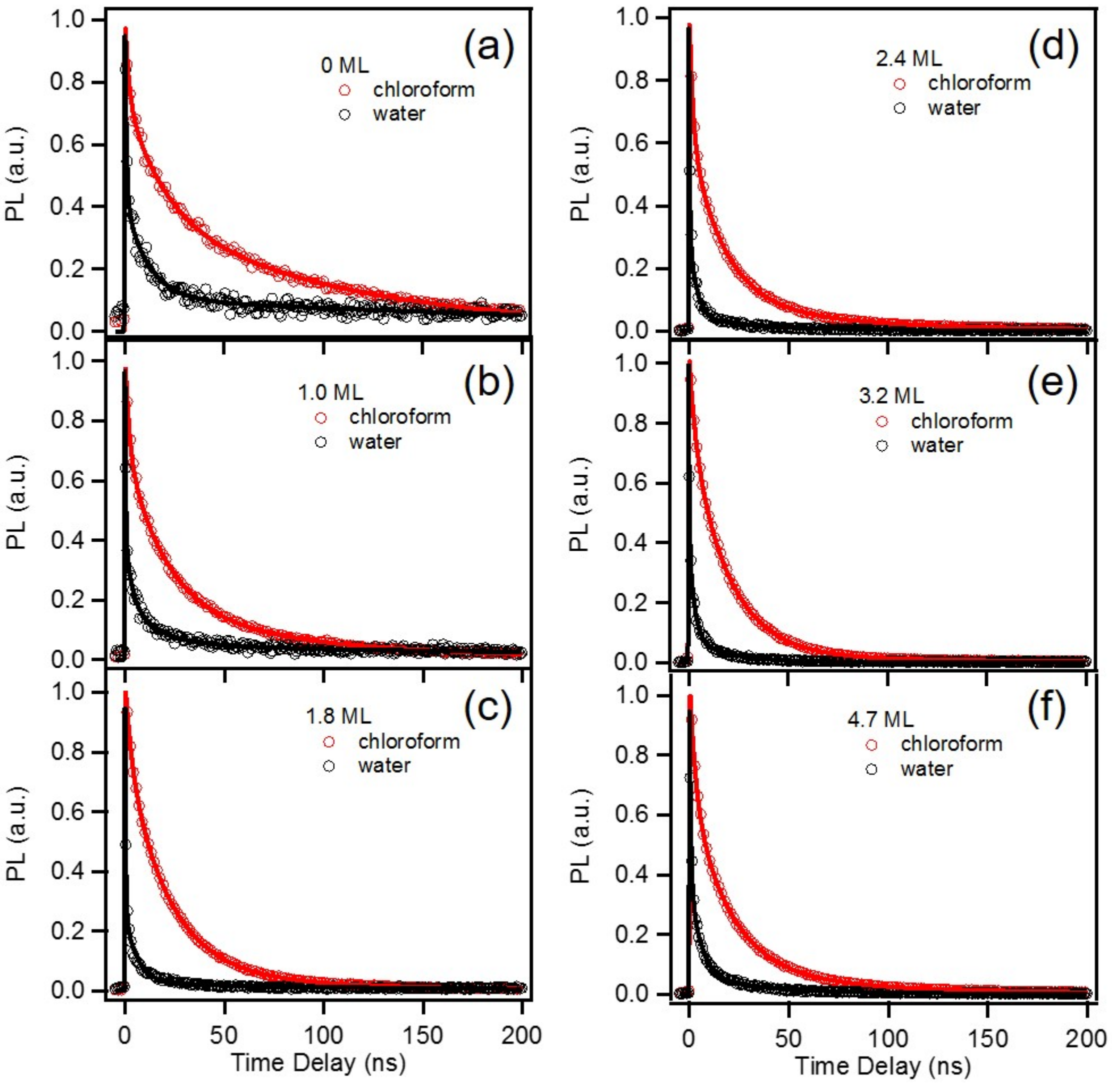

Figure S10. Comparison of PL decay of CdSe QDs with (a) 0, (b) 1.0, (c) 1.8, (d) 2.4, (e) 3.2 and (f) 4.7 MLs of CdS shells between chloroform and aqueous solutions after 400 nm excitation. (Solid red and black lines were the best fit)

SI 12. Comparison of time-resolved PL decay and 1S bleach TA signals of TOP-capped QDs in chloroform.

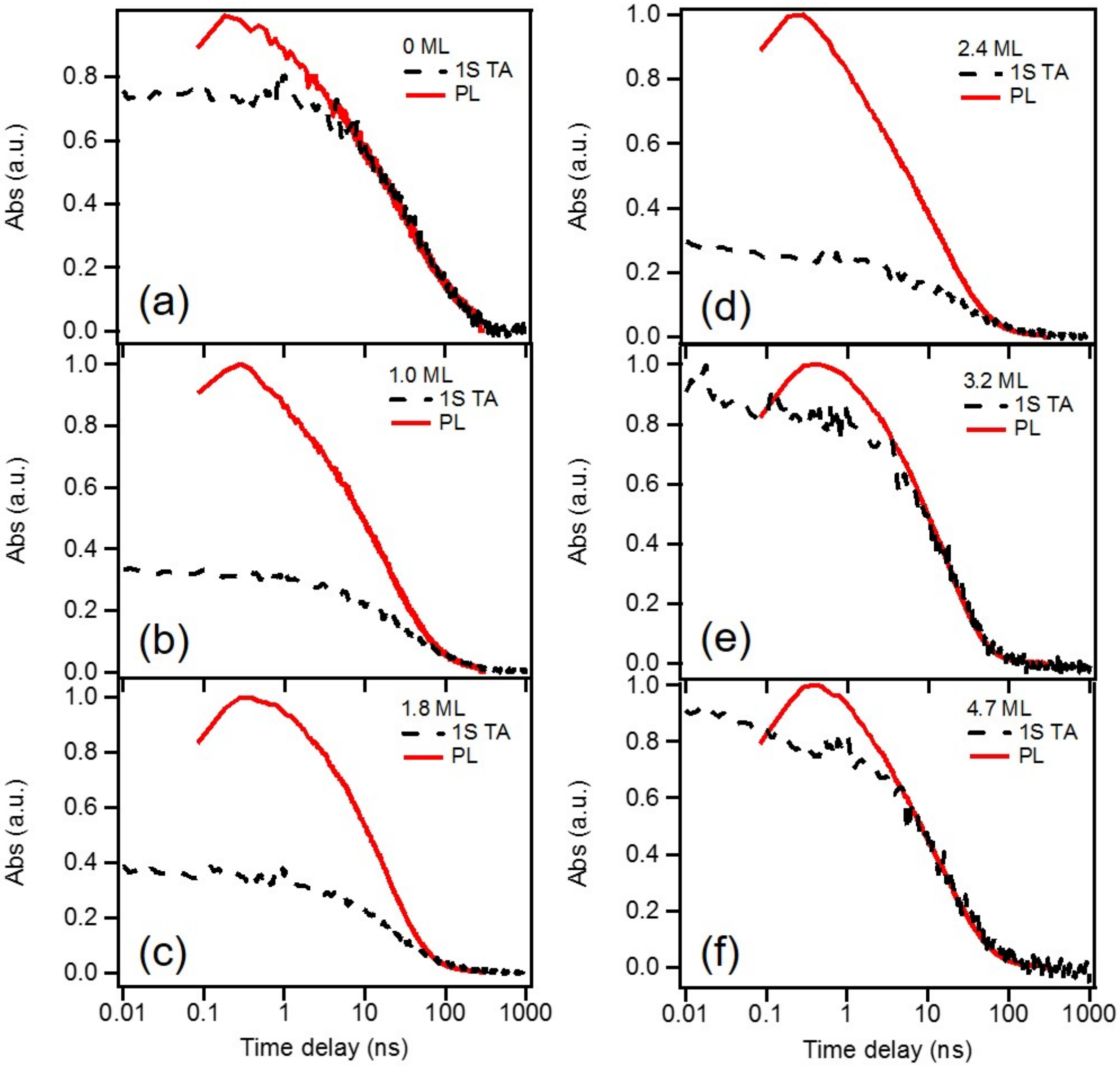

Figure S11. Comparison of time-resolved PL decay and 1S bleach TA signals of TOP-capped CdSe QDs with (a) 0, (b) 1.0, (c) 1.8, (d) 2.4, (e) 3.2 and (f) 4.7 MLs of CdS shells in chloroform. TA signals have been converted to positive and normalized to match the PL signals at detail for better comparison.

SI 13. Radial distribution function of 1S conduction-band electron and valence-band hole of quasi-type II CdSe/CdS quantum dots.

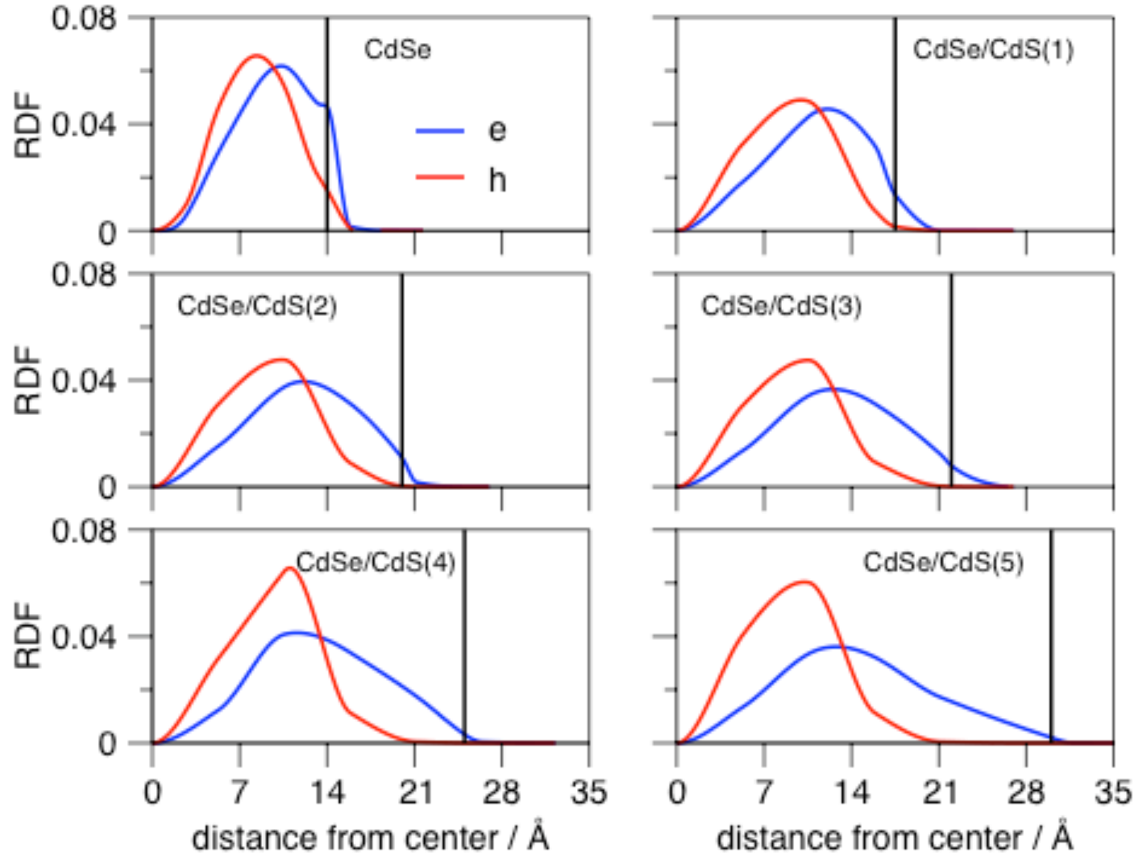

Figure S12. Radial distribution function of lowest energy (1s) conduction-band electron (solid red lines) and valence-band hole (solid blue lines) levels of quasi-type II CdSe/CdS quantum dots listed in Table S1. (vertical solid lines indicate shell/ligand interfaces)

SI 14. Fitting parameters of 1S exciton band in free CdSe/CdS quantum dots in chloroform and aqueous solutions.

Table S2A. 1S exciton bleach kinetics of TOP-capped CdSe/CdS QDs in chloroform. [a]

	0 ML	1.0 ML	1.8 ML	2.4 ML	3.2 ML	4.7 ML
A_1	-0.06	-0.08	-0.08	-0.15	-0.10	-0.10
τ_1 (ns)	0.02 ± 0.01	0.02 ± 0.01	0.02 ± 0.01	0.05 ± 0.01	0.10 ± 0.02	0.10 ± 0.02
A_2	-0.33	-0.50	-0.55	-0.46	-0.70	-0.47
τ_2 (ns)	16 ± 0.7	17 ± 0.4	14 ± 0.4	11 ± 0.4	13 ± 0.4	8.7 ± 0.4
A_3	-0.61	-0.42	-0.37	-0.39	-0.20	-0.43
τ_3 (ns)	88 ± 2	100 ± 2	77 ± 2	79 ± 2	68 ± 2	43 ± 2
$\tau_{1/2}$ (ns)	28.2	19.0	13.3	11.1	10.2	9.6

[a] The kinetic traces are fitted to the following equation: $\Delta A(t) = \sum_{i=1}^{3} A_i e^{-t/\tau_i}$

Table S2B. 1S exciton bleach kinetics of MUA-capped CdSe/CdS QDs in water.

	0 ML	1.0 ML	1.8 ML	2.4 ML	3.2 ML	4.7 ML
A_1	-0.12	-0.13	-0.17	-0.22	-0.25	-0.30
τ_1 (ns)	0.02 ± 0.01	0.02 ± 0.01	0.03 ± 0.01	0.05 ± 0.01	0.3 ± 0.1	0.3 ± 0.1
A_2	-0.61	-0.45	-0.36	-0.39	-0.58	-0.48
τ_2 (ns)	2.7 ± 0.1	1.1 ± 0.3	1.4 ± 0.1	1.9 ± 0.2	11 ± 0.3	5.9 ± 0.5
A_3	-0.27	-0.42	-0.47	-0.39	-0.17	-0.22
τ_3 (ns)	23 ± 1	16 ± 1	16 ± 1	28 ± 2	58 ± 4	41 ± 2
$\tau_{1/2}$ (ns)	2.4	1.6	2.5	2.5	3.1	3.0

SI 15. Fitting parameters of electron transfer rate of CdSe/CdS-MV^{2+} 1S exciton band kinetics.

Table S3. 1S exciton bleach kinetics of MUA-capped CdSe/CdS-MV^{2+} in aqueous solutions. [a]

	0 ML	1.0 ML	1.8 ML	2.4 ML	3.2 ML	4.7 ML
A_1	-0.86	-0.87	-0.63	-0.94	-0.61	-0.59
τ_1 (ps)	0.29 ± 0.06	0.20 ± 0.15	1.4 ± 0.1	1.6 ± 0.1	4.6 ± 0.3	17 ± 1
A_2	-0.19	-0.17	-0.43	-0.08	-0.44	-0.49
τ_2 (ps)	1.0 ± 0.3	1.6 ± 0.2	7.8 ± 0.7	16 ± 3	48 ± 2	85 ± 5
A_3	0.05	0.04	0.06	0.02	0.05	0.08
$\tau_{1/2}$ (ps)	0.3	0.6	1.5	1.7	6.3	20

[a] The kinetic traces solutions are fitted to the following equation:

$$\Delta A(t) = A_1 e^{-t/\tau_1} + A_2 e^{-t/\tau_2} + A_3$$

SI 16. Fitting parameters of average number (*m*) of adsorbed MV^{2+} molecules for 1.8 and 4.7 ML CdSe/CdS QDs.

Table S4A. 1S exciton bleach kinetics of CdSe/CdS QDs in aqueous solutions [a]

	**1.8 ML**	**4.7 ML**
**A_1 (mOD)**	-0.6	-1.0
**τ_1 (ps)**	5.65 ± 0.26	192 ± 10
**A_2 (mOD)**	-5.9	-6.6

[a] Fitting function is: $\Delta A(t) = (A_1 e^{-t/\tau_1} + A_2)$

Table S4B. 1S exciton bleach kinetics of CdSe/CdS QD-MV^{2+} in aqueous solutions [b]

**1.8 ML**		**4.7 ML**	
m	**k_{int} (10^{12} s^{-1})**	***M***	**k_{int} (10^{10} s^{-1})**
0.21 ± 0.02	1.24 ± 0.15	0.14± 0.01	7.50 ± 1.32
0.38 ± 0.02		0.36 ± 0.02	
0.52 ± 0.02		0.48 ± 0.02	
0.59 ± 0.02		0.55 ± 0.02	
0.69 ± 0.02		0.59 ± 0.02	
0.77 ± 0.03		0.65 ± 0.03	
0.78 ± 0.03		0.65 ± 0.02	

[b] Fitting function is $\Delta A(t) = (A_1 e^{-t/\tau_1} + A_2)(e^{m[e^{-k_{int}t}-1]})$ where τ_1 is kept to the same value listed in Table S4A.

SI 17. Fitting parameters of Langmuir isotherm to the mean surface coverage and MV^{2+} concentration.

Table S5. Fitting parameters of Langmuir isotherm [a]

	K_0 (M^{-1})	A_0
**1.8 ML**	19000 ± 2000	0.9 ± 0.1
**4.7 ML**	21000 ± 2000	0.8 ± 0.1

[a] The fitting function is Equation S3 in SI.

SI 18. Fitting parameters of MV^{2+} formation and decay kinetics.

Table S6. Fitting parameters of MV^{2+} formation and decay kinetics [a]

	0 ML	1.0 ML	1.8 ML	2.4 ML	3.2 ML	4.7 ML
A_1	-0.96	-0.98	-1.0	-0.99	-0.96	-0.95
τ_1 (ps)	0.48 ± 0.05	0.16 ± 0.01	0.27 ± 0.12	0.14 ± 0.02	3.3 ± 1.0	30 ± 3
A_2	0.23	0.19	0.25	0.52	0.10	0.41
τ_2 (ns)	0.03 ± 0.01	0.02 ± 0.01	0.12 ± 0.02	2.1 ± 0.3	0.53 ± 0.03	27 ± 2
A_3	0.59	0.50	0.60	0.21	0.66	0.30
τ_3 (ns)	0.76 ± 0.04	0.38 ± 0.03	2.2 ± 0.3	22 ± 5	20 ± 2	281 ± 50
A_4	0.10	0.19	0.09	0.15	0.13	0.20
τ_4 (ns)	18 ± 2	16 ± 1	18 ± 3	220 ± 20	290 ± 40	1080 ± 140
A_5	0.04	0.10	0.06	0.11	0.08	0.04
$\tau_{1/2}$ (ns)	0.5	0.6	1.2	4.4	17	100

[a] The kinetic traces solutions are fitted to the following equation:

$$\Delta A(t) = A_1 e^{-t/\tau_1} + A_2 e^{-t/\tau_2} + A_3 e^{-t/\tau_3} + A_4 e^{-t/\tau_4} + A_5$$

Here τ_1 is MV^{2+} radical formation while τ_2 to τ_4 are radical decay.

SI 19. Fitting parameters of time-resolved photoluminescence (PL) decay of CdSe/CdS quantum dots in aqueous solutions and in chloroform.

Table S7. PL decay of CdSe/CdS quantum dots in aqueous solutions and in chloroform [a]

	0 ML	1.0 ML	1.8 ML	2.4 ML	3.2 ML	4.7 ML
			**In chloroform**			
A_1	0.26	0.32	0.22	0.24	0.37	0.41
τ_1 (ns)	2.0 ± 0.3	2.0 ± 0.1	3.1 ± 0.2	3.1 ± 0.1	3.0 ± 0.1	1.7 ± 0.1
A_2	0.38	0.50	0.75	0.74	0.61	0.55
τ_2 (ns)	19 ± 1	20 ± 1	20 ± 1	19 ± 1	19 ± 1	17 ± 2
A_3	0.36	0.18	0.03	0.02	0.02	0.04
τ_3 (ns)	105 ± 2	89 ± 2	82 ± 1	91 ± 3	79 ± 2	72 ± 2
$\tau_{1/2}$ (ns)	15.6	9.3	11.3	10.4	9.8	8.1
			**In aqueous solutions**			
A_1	0.69	0.64	0.73	0.77	0.71	0.74
τ_1 (ns)	0.78 ± 0.02	0.37 ± 0.03	0.40 ± 0.01	0.33 ± 0.02	0.67 ± 0.05	0.34 ± 0.04
A_2	0.23	0.24	0.24	0.18	0.24	0.19
τ_2 (ns)	11 ± 1	16 ± 2	6.2 ± 0.2	2.8 ± 0.3	5.5 ± 0.5	3.7 ± 0.1
A_3	0.08	0.12	0.03	0.05	0.05	0.07
τ_3 (ns)	290 ± 10	280 ± 10	140 ± 10	20 ± 2	41 ± 1	41 ± 1
$\tau_{1/2}$ (ns)	1.3	0.6	0.4	0.4	0.5	0.9

[a] The kinetic traces solutions are fitted to the following equation:

$$\Delta A(t) = A_1 e^{-t/\tau_1} + A_2 e^{-t/\tau_2} + A_3 e^{-t/\tau_3}$$

SI 20. Theoretical methods.

Computational methods

A. The eigenvalue equation

In the present work we consider only the envelope part of the full particle wavefunction and limit the number of electrons, and holes, respectively to one and consider only the case of the lowest energy orbital occupation for each type of particle. For the spherical quantum dots considered here this amounts to the ground state $1S_e$ (for electron) and $1S_h$ (for hole) orbital occupations. Further, we ignore electron-hole exchange at this stage due to it being considerably smaller than the Coulomb interactions.[10] These conditions allow us to write an exciton wavefunction as a Hartree product of single particle wavefunctions (orbitals) and simplify the eigenvalue equations.

In the effective mass approximation,[11,12] the single-band, coupled Hartree equations for the electron, and the hole respectively, are given by

$$\left\{\vec{\nabla}\cdot\frac{-\hbar^2}{2m_e^*(\mathbf{r})}\vec{\nabla}+V_e(\mathbf{r})-\Phi_e^{\mathrm{C}}(\mathbf{r})\right\}\psi_e(\mathbf{r})=E_e\psi_e(\mathbf{r}), \tag{1a}$$

$$\left\{\vec{\nabla}\cdot\frac{-\hbar^2}{2m_h^*(\mathbf{r})}\vec{\nabla}+V_h(\mathbf{r})+\Phi_h^{\mathrm{C}}(\mathbf{r})\right\}\psi_h(\mathbf{r})=E_h\psi_h(\mathbf{r}) \tag{1b}$$

where $m_e^*(\mathbf{r})$ and $m_h^*(\mathbf{r})$ are the effective masses, $V_e(\mathbf{r})$ and $V_h(\mathbf{r})$ are the conduction and valence band levels, and $\Phi_e^{\mathrm{C}}(\mathbf{r})$ is the Coulomb potential acting on the electron, and $\Phi_h^{\mathrm{C}}(\mathbf{r})$ is the corresponding one acting on the hole. The eigenfunctions $\psi_e(\mathbf{r})$ and $\psi_h(\mathbf{r})$ are the *envelope* wavefunctions of the electron and hole, respectively, with the eigenvalues E_e and E_h. Quantum confinement energies are thus defined as $\Delta E_e = E_e - V_e$ and $\Delta E_h = V_h - E_h$ for electron and hole respectively, where V_e and V_h are conduction and valence band positions.

The eigenvalue equations are solved in a self-consistent manner by updating the Coulomb potential each time the wavefunctions are adjusted. The microiterations are carried out using Davidson's subspace expansion.[13] The Hamiltonian and the wavefunction are represented by a contracted discrete variable representation method as described previously.[14] We construct a uniform Cartesian grid with $\Delta x = \Delta y = \Delta z$ and retain the points within a cutoff radius of a quantum dot's center, R_c. See also Tables S8 and S9 for calculation details.

Table S8. Material parameters used in the calculations. m_0 is the electron mass in vacuum, e_0 is the dielectric constant of vacuum, and V_e and V_h are the conduction and valence band edges respectively.

	CdSe	CdS	chroloform
m_e^* / m_0	0.13	0.21	1
m_h^* / m_0	0.45	0.8	1
e / e_0	10	8.9	4.7
V_e / eV	-4.0	-3.78	0
V_h / eV	-5.7	-6.29	-8.4

B. Electrostatic potentials

In core-shell quantum structures embedded in a medium with a different dielectric constant, the Coulomb potential entering Eq. 1 generally consists of two parts: the *self-induced* polarization potential and the cumulative potential due to the other particles.[15,16] Specifically, for an electron-hole system the potentials are,

$$\Phi_e^{\mathrm{C}}(\mathbf{r}) = \frac{1}{2}\Phi_e^{\mathrm{ind}}(\mathbf{r}) + \Phi_h(\mathbf{r}), \qquad (2a)$$

$$\Phi_h^{\mathrm{C}}(\mathbf{r}) = \frac{1}{2}\Phi_h^{\mathrm{ind}}(\mathbf{r}) + \Phi_e(\mathbf{r}) \qquad (2b)$$

with the first term being the self-induced surface polarization potential,[17] due to a dielectric mismatch at an interface (in homogeneous dielectric media there is no induction). The second term arises due to the charge density of the other particle and an induced surface polarization due to that charge density. This source potential satisfies the Poisson equation,[18]

$$\vec{\nabla}\cdot\varepsilon(\mathbf{r})\vec{\nabla}\Phi_e(\mathbf{r}) = 4\pi\left|\psi_e(\mathbf{r})\right|^2, \text{ (3a)}$$

$$\vec{\nabla}\cdot\varepsilon(\mathbf{r})\vec{\nabla}\Phi_h(\mathbf{r}) = -4\pi\left|\psi_h(\mathbf{r})\right|^2 \text{ (3b)}$$

where $\varepsilon(\mathbf{r})$ is the position dependent dielectric constant. These equations are solved simultaneously with Eq. 1 using the same grid representation.[14] The solutions to Eq. 3 are matched with the known outer potential, $\varepsilon_{\mathrm{env}}^{-1}\int d\mathbf{r}'\,|\psi(\mathbf{r}')|^2/|\mathbf{r}'-\mathbf{r}|$, at the boundary defined by the surface $x^2+y^2+z^2=R_{\mathrm{c}}^2$ where the wavefunction is made to vanish (see above).

The induced potential is derived from the source potential by integrating the induced surface polarization density over all interfaces S which have a dielectric discontinuity,[18]

$$\Phi_e^{\text{ind}}(\mathbf{r}) = \frac{1}{4\pi}\sum_S \frac{\varepsilon_{out}^S - \varepsilon_{in}^S}{\varepsilon_{out}^S} \oint \frac{\vec{\nabla}\Phi_e(\mathbf{r})\big|_{in} \cdot \hat{n}_{out}^{in}(\mathbf{r}_S)}{|\mathbf{r} - \mathbf{r}_S|} dA_S\,, \qquad (4a)$$

$$\Phi_h^{\text{ind}}(\mathbf{r}) = \frac{1}{4\pi}\sum_S \frac{\varepsilon_{out}^S - \varepsilon_{in}^S}{\varepsilon_{out}^S} \oint \frac{\vec{\nabla}\Phi_h(\mathbf{r})\big|_{in} \cdot \hat{n}_{out}^{in}(\mathbf{r}_S)}{|\mathbf{r} - \mathbf{r}_S|} dA_S\,. \qquad (4b)$$

The integrals are calculated numerically by casting the surface integral into a volume integral and integrating over the grid points falling inside the inner shell and using an analytic expression for electric potential gradient (see Appendix). By the convention used in deriving Eq. 4 the dielectric constants ε_{in}^S and ε_{out}^S are measured on either side of the interface along the surface norm pointing from inside to outside $\hat{n}_{out}^{in}$, and the potential gradient is evaluated just inside the interfacial surface. We also used the fact that the electric field $\vec{F}$, crossing interface S along the normal direction, has a discontinuity: $\varepsilon_{in}^S \vec{F}_{in}^S \cdot \hat{n} = \varepsilon_{out}^S \vec{F}_{out}^S \cdot \hat{n}$. We note that others have used analytic forms of Eq. 4 for the special case of a spherical quantum dot with a single interface and infinite square potential.[19]

To sum up, in an electron-hole system, i.e. a neutral exciton confined in a quantum dot, the electron feels (*i*) interaction with its own image across an interface $W_{e-(e)}$, (*ii*) interaction with the hole W_{e-h}, (the latter is often called exciton binding energy) and (*iii*) interaction with hole's image across the same interface, $W_{e-(h)}$. To calculate these quantities we need to integrate the corresponding potentials over the density,

$$W_{\text{e-(e)}} = -\frac{1}{2}\int d\mathbf{r}\Phi_e^{\text{ind}}(\mathbf{r})\left|\psi_e(\mathbf{r})\right|^2, \tag{5a}$$

$$W_{\text{h-(h)}} = \frac{1}{2}\int d\mathbf{r}\Phi_h^{\text{ind}}(\mathbf{r})\left|\psi_h(\mathbf{r})\right|^2, \tag{5b}$$

$$W_{\text{e-(h)}} = -\int d\mathbf{r}\Phi_h^{\text{ind}}(\mathbf{r})\left|\psi_e(\mathbf{r})\right|^2, \tag{5c}$$

$$W_{\text{h-(e)}} = \int d\mathbf{r}\Phi_e^{\text{ind}}(\mathbf{r})\left|\psi_h(\mathbf{r})\right|^2, \tag{5d}$$

where the factor of 1/2 in the self-image terms corrects for overcounting.[17] Exciton binding is defined as the sum of the full particle energy in the presence of electric field and other particle self-energy,

$$W_{\text{e-h}} = -\int d\mathbf{r}\Phi_e^{\text{C}}(\mathbf{r})\left|\psi_e(\mathbf{r})\right|^2 + W_{\text{h-(h)}} = \int d\mathbf{r}\Phi_h^{\text{C}}(\mathbf{r})\left|\psi_h(\mathbf{r})\right|^2 + W_{\text{e-(e)}}, \tag{6a}$$

or in equivalent form

$$W_{\text{e-h}} = W_{\text{(e-h)}} + W_{\text{e-(e)}} + W_{\text{h-(h)}}, \tag{6b}$$

where

$$W_{(e-h)} = -\int d\mathbf{r}\Phi_h(\mathbf{r})|\psi_e(\mathbf{r})|^2 = \int d\mathbf{r}\Phi_e(\mathbf{r})|\psi_h(\mathbf{r})|^2 \quad (7)$$

is the energy due to the other particle and its image. In most cases involving spherical Type 1 quantum dots, the two image interactions are expected to approximately cancel each other due to very similar charge distributions, thus, the exciton binding energy consists mainly of the direct interaction with the hole as if no induced polarization were present.[17]

The quantum dot band gap, or the 1S-1S transition, is calculated from the confinement energy,

$$E_{1S-1S} = E_g + \Delta E_e + \Delta E_h - W_{(e\text{-}h)} \quad (8)$$

where E_g is the bulk band gap.

CdSe/CdS quantum dot calculations

For the calculations reported here we use Type 1 CdSe/ZnS core/shell quantum dots. The CdSe core radius is 1.4 nm, and CdS shell thickness varies from 1 to 5 monolayers, with a monolayer thickness varying from ~0.2 to 0.5 nm. Material parameters and dot sizes are given in Tables 1 and 2. The number of retained grid points, which is the size of the DVR

matrix in the eigenvalue and the Poisson equations, is determined by both the cut-off radius R_c and the grid resolution $\Delta x = \Delta y = \Delta z$.

Table S9. Quantum dot sizes and grid parameters.

Seed/Shell sample	radius / nm	shell thickness / nm	cut-off radius, R_c / nm	grid resolution, Δx / bohr	retained grid points
CdSe seed	1.4	0	2.4	2.0	48885
CdSe/CdS-1	1.75	0.35	2.75	2.2	55179
CdSe/CdS-2	2	0.6	3	2.2	71711
CdSe/CdS-3	2.2	0.8	3.2	2.2	87079
CdSe/CdS-4	2.5	1.1	3.5	2.8	55179
CdSe/CdS-5	3	1.6	4	3.2	55179

The results are summarized in Table S10. The confinement energies for the electron and the hole are understandably quite different in magnitude due to the difference in effective mass. The trend in the confinement energy with shell thickness suggests that the electron 1S wavefunction extends out into the shell region while the hole 1S remains localized within the seed. This is the key difference shaping the trend in the charge separation and recombination rates, as functions of shell thickness, addressed in the main text.

We note that the electrostatic interactions follow the expected behavior. Both the self-image energies and other-image energies are similar in magnitude for electron and hole. The hole feels a slightly stronger self-repulsion than the electron does, while the electron feels a slightly stronger attraction to hole's image than does the hole to electron's image. This may be explained by the more localized hole density, which does not penetrate significantly into the shell region. The sum of two self-image energies approximately cancels other-particle-image energy, which is another indication that in the present spherical quantum dots the electron-hole Coulomb energy is almost entirely due to the direct interaction. We can verify this by calculating the approximate binding energy using an average dielectric constant,

$$\tilde{W}_{\mathrm{e-h}} = \iint d\mathbf{r}_e\, d\mathbf{r}_h \frac{2}{\varepsilon(\mathbf{r}_e) + \varepsilon(\mathbf{r}_h)} \frac{\rho_e(\mathbf{r}_e)\rho_h(\mathbf{r}_h)}{\left|\mathbf{r}_e - \mathbf{r}_h\right|}. \tag{9}$$

The approximate binding energies are remarkably close to the exact ones especially in the large dots where both particle densities are far removed from the interface with the biggest dielectric mismatch (CdS/chloroform).

Table S10. Confinement energy *DE*, direct Coulomb energy $W_{e\text{-}h}$, and its approximation in parantheses (see Eq 9), self-image interaction energies $W_{e\text{-}(e)}$, $W_{h\text{-}(h)}$, other-image energies $W_{e\text{-}(h)}$, $W_{h\text{-}(e)}$ in meV, and $1S_e$-$1S_h$ transition energy $E_{1S\text{-}1S}$ in eV.

Seed/Shell	DE_e	DE_h	$W_{e\text{-}h}$ ($\tilde{W}_{e-h}$)	$W_{e\text{-}(e)}$	$W_{h\text{-}(h)}$	$W_{e\text{-}(h)}$	$W_{h\text{-}(e)}$	$E_{1S\text{-}1S}$
CdSe seed	408	98	-141 (-144)	56	61	-120	-112	2.463
CdSe/CdS-1	285	68	-102 (-127)	58	61	-121	-115	2.274
CdSe/CdS-2	230	63	-118 (-119)	40	43	-86	-81	2.194
CdSe/CdS-3	207	71	-110 (-114)	39	42	-83	-78	2.168
CdSe/CdS-4	186	81	-95 (-108)	39	42	-84	-79	2.144
CdSe/CdS-5	161	87	-103 (-101)	27	30	-59	-55	2.108

Appendix

It can be shown that for a grid contracted down from an infinite expansion (as is done in the present work), the solution to Eqs. 1 and 3, in general, has a simple analytic representation,[14,20]

$$\Phi(\mathbf{r}) = \sum_{ijk} \Phi_{ijk} \ \mathrm{sinc}(\pi \tilde{x}_i) \ \mathrm{sinc}(\pi \tilde{y}_j) \ \mathrm{sinc}(\pi \tilde{z}_k) \quad \text{(A1)}$$

where in the present case Φ_{ijk} is the discrete solution of Eq. 3, $\tilde{x}_i \equiv (x - x_i)/\Delta x$, etc, and the sum runs over the entire grid. The derivative of Eq. A1 at grid point $i'j'k'$ readily follows. Noting that $\mathrm{sinc}(0) = 1$ and $\mathrm{sinc}(n\pi) = 0$ for any integer $n \neq 0$, we obtain

$$\left.\frac{\partial \Phi}{\partial x}\right|_{i'j'k'} = \frac{\pi}{\Delta x} \sum_{i \neq i'} \Phi_{ij'k'} \frac{\cos(\pi (x_{i'} - x_i)/\Delta x)}{\pi (x_{i'} - x_i)/\Delta x}, \qquad \text{(A2a)}$$

$$\left.\frac{\partial \Phi}{\partial y}\right|_{i'j'k'} = \frac{\pi}{\Delta y} \sum_{j \neq j'} \Phi_{i'jk'} \frac{\cos(\pi (y_{j'} - y_j)/\Delta y)}{\pi (y_{j'} - y_j)/\Delta y}, \qquad \text{(A2b)}$$

$$\left.\frac{\partial \Phi}{\partial z}\right|_{i'j'k'} = \frac{\pi}{\Delta z} \sum_{k \neq k'} \Phi_{i'j'k} \frac{\cos(\pi (z_{k'} - z_k)/\Delta z)}{\pi (z_{k'} - z_k)/\Delta z}. \qquad \text{(A2c)}$$

These quantities are obtained on the fly at a fraction of the total cost of the entire procedure and are used to evaluate Eqs 4.

SI 21. Reference

(1) Carbone, L.; Nobile, C.; De Giorgi, M.; Sala, F. D.; Morello, G.; Pompa, P.; Hytch, M.; Snoeck, E.; Fiore, A.; Franchini, I. R. *Nano Lett.* 2007, *7*, 2942.

(2) Li, J. J.; Wang, Y. A.; Guo, W.; Keay, J. C.; Mishima, T. D.; Johnson, M. B.; Peng, X. *J. Am. Chem. Soc.* 2003, *125*, 12567.

(3) van Embden, J.; Jasieniak, J.; Mulvaney, P. *J. Am. Chem. Soc.* 2009, *131*, 14299.

(4) Issac, A.; Jin, S.; Lian, T. *J. Am. Chem. Soc.* 2008, *130*, 11280.

(5) Song, N.; Zhu, H.; Jin, S.; Zhan, W.; Lian, T. *ACS nano* 2010, *5*, 613.

(6) Jin, S.; Hsiang, J.-C.; Zhu, H.; Song, N.; Dickson, R. M.; Lian, T. *Chem. Sci.* 2010, *1*, 519.

(7) Boulesbaa, A.; Issac, A.; Stockwell, D.; Huang, Z.; Huang, J.; Guo, J.; Lian, T. *J. Am. Chem. Soc.* 2007, *129*, 15132.

(8) Huang, J.; Stockwell, D.; Huang, Z. Q.; Mohler, D. L.; Lian, T. Q. *J. Am. Chem. Soc.* 2008, *130*, 5632.

(9) Morris-Cohen, A. J.; Frederick, M. T.; Cass, L. C.; Weiss, E. A. *J. Am. Chem. Soc.* 2011, *133*, 10146.

(10) Franceschetti, A.; Zunger, A. *Phys. Rev. Lett.* 1997, *78*, 915.

(11) Brus, L. E. *J. Chem. Phys.* 1984, *80*, 4403.

(12) Brus, L. *J. Chem. Phys.* 1983, *79*, 5566.

(13) Davidson, E. R. *Journal of Computational Physics* 1975, *17*, 87.

(14) Kaledin, A. L.; Lian, T.; Hill, C. L.; Musaev, D. G. *Journal of Chemical Theory and Computation* 2014, *10*, 3409.

(15) Saravanamoorthy, S.; Peter, A. J.; Lee, C. W. *Physica E: Low-dimensional Systems and Nanostructures* 2014, *63*, 337.

(16) Piryatinski, A.; Ivanov, S. A.; Tretiak, S.; Klimov, V. I. *Nano Lett.* 2007, *7*, 108.

(17) Allan, G.; Delerue, C.; Lannoo, M.; Martin, E. *Phys. Rev. B* 1995, *52*, 11982.

(18) Jackson, J. D.; Jackson, J. D. *Classical electrodynamics*; Wiley New York etc., 1962; Vol. 3.

(19) Cristea, M.; Niculescu, E. *The European Physical Journal B-Condensed Matter and Complex Systems* 2012, *85*, 1.

(20) Colbert, D. T.; Miller, W. H. *J. Chem. Phys.* 1992, *96*, 1982.
